# Empirical Evaluation of Inflorescences’ Morphological Attributes for Yield Optimization of Medicinal Cannabis Cultivars

**DOI:** 10.3389/fpls.2022.858519

**Published:** 2022-04-19

**Authors:** Erez Naim-Feil, Edmond J. Breen, Luke W. Pembleton, Laura E. Spooner, German C. Spangenberg, Noel O. I. Cogan

**Affiliations:** ^1^Agriculture Victoria, AgriBio, Centre for AgriBioscience, Melbourne, VIC, Australia; ^2^School of Applied Systems Biology, La Trobe University, Melbourne, VIC, Australia

**Keywords:** breeding, plant productivity, heritability, selection, image analysis, production

## Abstract

In recent decades with the reacknowledgment of the medicinal properties of *Cannabis sativa* L. (cannabis) plants, there is an increased demand for high performing cultivars that can deliver quality products for various applications. However, scientific knowledge that can facilitate the generation of advanced cannabis cultivars is scarce. In order to improve cannabis breeding and optimize cultivation techniques, the current study aimed to examine the morphological attributes of cannabis inflorescences using novel image analysis practices. The investigated plant population comprises 478 plants ascribed to 119 genotypes of high−THC or blended THC−CBD ratio that was cultivated under a controlled environment facility. Following harvest, all plants were manually processed and an image of the trimmed and refined inflorescences extracted from each plant was captured. Image analysis was then performed using in-house custom-made software which extracted 8 morphological features (such as size, shape and perimeter) for each of the 127,000 extracted inflorescences. Our findings suggest that environmental factors play an important role in the determination of inflorescences’ morphology. Therefore, further studies that focus on genotype X environment interactions are required in order to generate inflorescences with desired characteristics. An examination of the intra-plant inflorescences weight distribution revealed that processing 75% of the plant’s largest inflorescences will gain 90% of its overall yield weight. Therefore, for the optimization of post-harvest tasks, it is suggested to evaluate if the benefits from extracting and processing the plant’s smaller inflorescences outweigh its operational costs. To advance selection efficacy for breeding purposes, a prediction equation for forecasting the plant’s production biomass through width measurements of specific inflorescences, formed under the current experimental methodology, was generated. Thus, it is anticipated that findings from the current study will contribute to the field of medicinal cannabis by improving targeted breeding programs, advancing crop productivity and enhancing the efficacy of post-harvest procedures.

## Introduction

For millennia, *Cannabis sativa* L. (cannabis) has been utilized by mankind as a multipurpose source for fiber and oil-seed products, as hemp, and for ritual, recreational and medicinal applications as cannabis (Abel, 1980; Small et al., 2003; Small, 2017a). The utilization of cannabis for medicinal purposes is well-documented since ancient times (Zuardi, 2006; Russo, 2014) but its breakthrough into the modern pharmacopoeia occurred only during the 19th century, when western physicians recognized its therapeutic potential (Mikuriya, 1969; Small, 2017a). However, due to advances in medicinal technology and alternative medication during the first half of the 20th century, the popularity of cannabis dwindled (Mikuriya, 1969; Hand et al., 2016; Small, 2017a) but concurrently, its recreational consumption became more prevalent (Hand et al., 2016). Over this period, international prohibition of cannabis and cannabis trafficking was initiated, labeling the cannabis plant and its products as narcotics (Erkelens and Hazekamp, 2014; Pain, 2015; Pisanti and Bifulco, 2017). As a result, scientific research on cannabis almost completely ceased (Sobo, 2017) and its medical use was diminished significantly (Mikuriya, 1969; Small, 2017a) whilst its illegal recreational use was widespread (Zuardi, 2006). In order to meet the illegal market demands, cannabis growers had to implement its cultivation under clandestine locations which necessitated plant adaptations to suboptimal growth conditions (Small, 2017a). These adaptations were successfully implemented through unofficial breeding initiatives (Clarke and Merlin, 2013; Barcaccia et al., 2020) and cannabis has become one of the most extensive and fast-growing illicit drug distributed and consumed worldwide (Hall and Degenhardt, 2007). Hence, while other commercial crops were being scientifically evaluated and improved (Godfray et al., 2010), cannabis, due to its illicit status, was not examined by advanced and contemporary scientific tools and therefore, its breeding potential is currently still in its infancy (Chouvy, 2019).

Since the turn of the century, the potential of medicinal cannabis has been scientifically reacknowledged through a large number of studies (Nahtigal et al., 2016). These have suggested that cannabis-based remedies can alleviate and treat a wide range of medical disorders (Cascio et al., 2017) such as nausea (Parker et al., 2002), psychotic symptoms of schizophrenia (Leweke et al., 2012), pediatric epilepsy (Goldstein, 2015) and pain (Baron, 2018). The pharmaceutical properties of cannabis are generally ascribed to the plant’s secondary metabolites and especially to the phytocannabinoids (cannabinoids) (Jin et al., 2020). To date, 120 different cannabinoids have been scientifically identified (ElSohly et al., 2017; Radwan et al., 2017) amongst them, Δ9-tetrahydrocannabinol (THC) and cannabidiol (CBD) are the most abundant, well-known and extensively studied due to their broad medicinal attributes (Cascio et al., 2017; Chandra et al., 2020). Cannabinoids can be found across most tissues of both male and female cannabis plants (Tanaka and Shoyama, 1999; Raman et al., 2017) but the most profuse cannabinoid concentration is found over inflorescences’ trichomes of pistillate plants (Potter, 2013; Thomas and ElSohly, 2015). The ovary of each floret that forms the inflorescence is surrounded by transparent perianth and green bracts which are considered to be the location of the most abundant trichome coverage within the cannabis plant (Hammond and Mahlberg, 1977). Due to the high cannabinoid concentration found over the plant’s inflorescences, they are utilized as the main commercial product of the medicinal cannabis industry (Radwan et al., 2017; Hawley et al., 2018). Morphologically, cannabis florets develop close to the plant’s stem in a sessile or subsessile structure and near to shoot apex, they aggregate together to form a continuous and congested inflorescence (Small, 2015; Raman et al., 2017).

Scientific research has targeted cannabis inflorescences and largely focused on microscopic aspects of the distribution, formation, structure and morphogenesis of trichomes (Hammond and Mahlberg, 1973; Dayanandan and Kaufman, 1976; Ebersbach et al., 2018; Livingston et al., 2020) as well as the biosynthesis pathways of secondary metabolites (Flores-sanchez and Verpoorte, 2008; Andre et al., 2016). However, in the last decade, the expanding market of medicinal cannabis has become a valuable industry that primarily relies on the cultivation and production of cannabis plant material (Small, 2017a; Summers, 2018; Parker et al., 2019) and therefore, there is now a strong focus on the optimization of cannabis yield in the whole-plant production level. Currently, one of the greatest challenges of this industry is to cultivate prolific and uniform plants which provide a consistent supply of homogenous plant material (Magagnini et al., 2018; Lata et al., 2019; Burgel et al., 2020). In response to this, several studies that were designed to improve plant productivity have examined the effect of key environmental factors such as exogenic growth regulators (Burgel et al., 2020), light source and light spectrum (Magagnini et al., 2018; Danziger and Bernstein, 2021a), stress (Caplan et al., 2019), plant architecture (Danziger and Bernstein, 2021b) and optimized fertilization regimes (Caplan et al., 2017; Bernstein et al., 2019a; Saloner and Bernstein, 2021) on plant yield. Although these studies have generated significant knowledge regarding the plant’s secondary metabolites and yield production, studies that characterize the inflorescence as a singular entity remain scarce. Usually, cannabis yield productivity is evaluated by the total weight of milled inflorescences (per plant or unit area) and the cannabinoid content in the harvested plant material (Magagnini et al., 2018). However, for breeding initiatives that focus on the enhancement of inflorescence biomass, using the weight indication solely is incomplete as there is an abundance of information concerning attributes of the intact inflorescences such as the number per plant, the distribution of size, uniformity, length and width, shape and perimeter that remains untapped. Although this knowledge can be beneficial for crop improvement, obtaining inflorescence data is challenging and requires a rigorous preparation process of manual separation from the plant, trimming of vegetative parts (in order to reflect genuine sizes) and measuring a wide distribution of phenotypic traits. To overcome the challenges with many of the manual aspects of data recording and generation, the current study employed image analysis techniques that are often used to evaluate plants’ characteristics (Samal et al., 2020) such as canopy growth (Kipp et al., 2014; Gage et al., 2017; Cai et al., 2018), plant architecture (Nguyen et al., 2015), seed attributes (Tanabata et al., 2012; Jahnke et al., 2016), and root characterization (Cai et al., 2015; Jeudy et al., 2016) for the assessment of inflorescence’s morphological attributes. It is plausible that uncovering the inflorescences’ morphological characteristics will advance breeders’ capability to generate cannabis plants with a tailored and desired yield morphology. These insights can potentially advance the cannabis industry by enhancing yield productivity through inflorescence size optimization, generating specific commodities that are in line with the industry requirements and increase labor efficacy during the processing of harvested plants. To the best of our knowledge, studies applying image analysis approaches for the quantification of intact inflorescences’ parameters have not been performed before.

The objective of this study is to characterize morphological inflorescence attributes that can optimize cannabis breeding and advance crop productivity. To address this goal we aim to (I) Characterize the morphological properties of cannabis inflorescences across various medicinal cannabis genotypes and estimate their variation and homogeneity within and between genotypes (II) Link the overall yield production and inflorescences’ features to identify key parameters for yield enhancement (III) Examine associations between inflorescences’ attributes and the plant’s physiological and phenological features.

## Materials and Methods

All work in the current study was carried out under the Medicinal Cannabis Research License (RL011/18) and Permits (RL01118P6 and RLO1118P3) issued by the Department of Health (DoH), Office of Drug Control (ODC), Australia.

A detailed description of the plants’ material used and the cultivation and physiological evaluation methods performed has been previously published (Naim-Feil et al., 2021). The following section will present a summarized version of the above and will expand on inflorescence evaluation techniques and image analysis methodologies.

### Plant Material and Trial Design

In the current study, a heterogeneous population of 119 cannabis genotypes was assessed under controlled environment (CE) conditions. Ninety-seven genotypes of the examined plant population have been imported as genetically unique seeds of medicinal cannabis cultivars from Canadian commercial companies. The remaining twenty-two genotypes have been developed by Agriculture Victoria Research as crosses between High-THC cannabis lines and high CBD accessions which contain a genetic background of an off-type hemp plant. Major cannabinoid profiles (THC and CBD) for all of the experimented genotypes were predicted through the B1080/1192 DNA marker (Pacifico et al., 2006). The molecular data indicated that the cannabinoid profile of the plant population was segregated into 2 groups of 74 and 45 genotypes, of high-THC:low-CBD and blended THC:CBD ratio, respectively.

The trial was designed in a randomized incomplete block design manner and 4–5 plant replicates of each genotype were examined in order to refine the effect of genetic factors on the phenotypic performance (Naim-Feil et al., 2021).

### Growth Conditions

Clonal replicates for each genotype were generated from a single mother plant that was maintained under long photoperiod (18 h light). Each mother plant was utilized to extract 10 cuttings, similar in size (10.5 ± 0.5 cm) and vigor. To invigorate root development, rooting hormone (Growth Technologies, Clonex, 3 g/L IBA gel, Perth, Australia) was applied at the base of each cutting before it was planted into coconut coir propagation plugs (Jiffy-7C,^®^ 50 mm, Zwijndrecht, Netherlands). Plant establishment took place under CE conditions with a 24/18°C (day/night) temperature regime, daylight cycle of 16/8 h (day/night), light intensity (PPFD) of 360 μmol m^–2^s^–1^ (measured on cultivating shelves surface, 35 cm below the light source) and relative humidity (RH) set to 55% in an indoor room. Thirty days after propagation, 6–7 cuttings, corresponding in size and vigor were selected for each genotype and transplanted into large coconut coir plugs (Jiffypots,^®^ ø8 cm, Zwijndrecht, Netherlands). Fourteen days later, 4–5 uniform plants of each genotype were selected and transplanted into coconut coir grow slabs (100 cm × 16 cm × 10 cm, Cazna grow slabs, Sydney, Australia) with 40 and 20 cm intervals between and within rows, respectively. The overall plant density within the CE cultivation facility was 4.3 plants × m^–2^. Plants were cultivated over drainage trays (Danish Hydro Trays 338 cm × 148 cm, Ringe, Denmark) that were situated on a rolling bench system. The vegetative growth phase lasted 42 days (from the date of transplanting) and the transition into the reproductive growth phase was induced by shortening the daylight cycle. Light intensity (PPF, wavelength range of 400–800 nm) of 2,150 μmol s^–1^ delivered by high-pressure sodium bulbs (Philips, MASTER GreenPower Xtra 1,000 W EL/5 × 6 CT, Amsterdam, Netherland), provided photoperiodic regimes of 18 and 12 h for the vegetative and the reproductive growth phases, respectively. Total fertigation of 1.3 liters × day^–1^ × plant^–1^ was delivered by a controlled drip irrigation system (Jain Octa-BubblerTM, 7.5 L/h, Fresno, California, United States), applying 1% A and B nutrient solution (THCTM, coco A + B, Melbourne, Australia) with an EC of 2.1 dS/m and pH levels of 6–6.1. Throughout the growing season, the temperature was maintained at 20°C/17°C (day/night) and the RH was set at 60%. To prevent breaking and overshading, wooden stakes were attached to tilted plants. Pest management was controlled by beneficial (arthropods) that were regularly distributed during the cultivation season.

### Data Recording and Plant Processing

**FIGURE 1:**
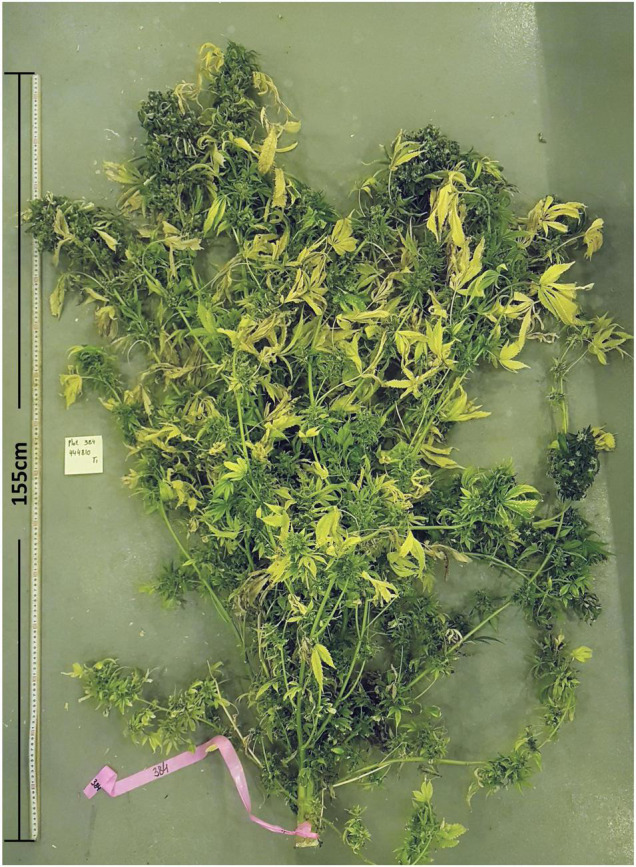
A whole cannabis plant at the time of harvest.

**FIGURE 2:**
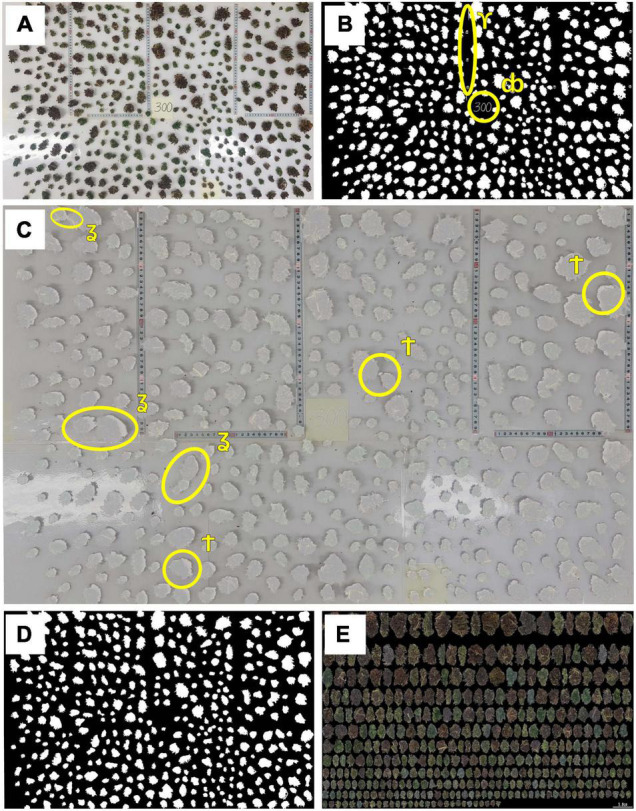
Preparation procedure for inflorescence image analysis. **(A)** An example of the total processed inflorescences distributed over a scaled surface which formed the raw image for morphological analysis,**(B)** Binary (black and white) image of the distributed inflorescences, **(C)** Raw and binary images juxtaposed onto one figure to highlight any requirements for manual editing, **(D)** Processed (cleaned) binary image with only inflorescence material included, **(E)** Composite image containing all inflorescences within the examined plant which are ordered by size. The following symbols indicate areas of concerns in the raw data: ϒ denotes a section with non-inflorescence material which needs to be removed;db indicates the location of plant index; ʓ shows areas of inflorescences contact which need to be separated and † indicates shaded areas to be removed which could be mistaken for inflorescence.

Physiological and phenological parameters have been recorded individually for each plant. Phenological assessment (Day to Maturation, DTM) was performed every 3 days during the flowering phase and defined as the period that lasted between the short daylight induction and the appearance of brown-shaded stigmas on 3 independent inflorescences. Plant harvest was performed selectively when ∼70% of the overall florets’ stigmas turned brown (Supplementary Figure 1A). The spatially adjusted genotypic mean of plant height (PH) range on harvest day spans from 95 to 157 cm with an overall mean of 122 cm (Naim-Feil et al., 2021). Harvested plants (Figure 1) were processed under both fresh (on harvest day) and dried (following drying) states. All plants were manually processed to separate inflorescences from vegetative substances (stems, foliage leaves) and the extracted inflorescences were further purified by removing vegetative organs (leaves and petioles) using leaves trimmers (Supplementary Figures 1B–D, Growlush^®^ bowl trimmer, 19″, Melbourne, Australia) in order to capture dependable and refined parameters. The processed inflorescences were then distributed over a scaled surface (Figure 2A) and photographed perpendicularly above the surface center to capture 12 MP images, with square pixels, *via* a dual-pixel camera (Samsung Galaxy S8, Seoul, South Korea). Large field image optical distortion was controlled by enabling the camera’s ultra-wide shape correction feature. Following this, inflorescence material was placed in a drying room (25°C, 20% humidity) for a minimum period of 14 days and the overall inflorescence dry biomass (IDB) was measured for each plant after complete dehydration was performed using a freeze dryer (VirTis, GPFD, Gardiner, NY, United States).

### Inflorescence Evaluation and Image Analysis Data Extraction

When needed, image editing was performed manually using GIMP (GIMP Development Team, 2.10.12, 2019) whereas image analysis for cannabis inflorescence shape identification and data extraction was carried out by in-house custom-made C++ (C++17 ISO standard compliant) software. A cm:pixel ratio was calculated independently for each image *via* measuring tapes that were embedded in the photo’s background (Figure 2A) to generate metric/absolute values for recorded parameters. Raw scaled-surface images were first converted into a binary format (black and white image, Figure 2B). As scaled-surface images were RGB color images, it was found that a combination of the blue band, from the RGB image, and the use of minimum error thresholding (Kittler and Illingworth, 1986) was a reliable and robust approach to automate the production of inflorescence binary images (Figure 2B) across all captured photographs. The initial binary images were further refined manually by constructing a juxtaposed figure (Figure 2C) using Gimp’s image layering that enabled manual elimination of undesired objects (e.g., remnant pieces of measurement scales and plant identification tags) and improvements to the segmented-image data by removing image shading or by separating any touching inflorescences that had not been correctly delimited by the software.

**FIGURE 3:**
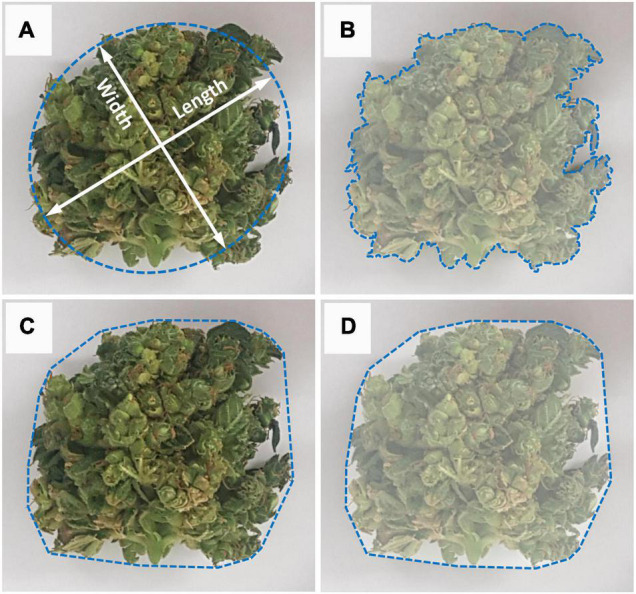
Measurement methodology for image analysis of the inflorescence’s morphological parameters. **(A)** Measurement of inflorescence length (major axis) and width (minor axis). Major axis defined as the longest line that can be drawn across the inflorescence and width defined as the longest line that can be drawn through the object while maintaining a perpendicular intersection with the major axis, **(B)** Inflorescence area is defined as the area enclosed within the object perimeter, **(C)** Convex perimeter (Hull Perimeter) measures the surrounding perimeter that contains the inflorescence object, **(D)** Convex hull is the area enclosed within the inflorescence’s convex perimeter.

These manually corrected binary images were then subject to further automated image editing, using image analysis, to remove isolated inflorescences/leaf material in each image having an area of less than 1 cm^2^, using area openings (Breen and Jones, 1996). All cleaned and processed binary images (Figure 2D) were then analyzed and inflorescence length (IL), inflorescence width (IW), convex hull (CH) area and hull perimeter (HP) of each object within each plant were recorded (Figure 3).

Inflorescence size (IS) was determined from the number of pixels making up the object. The length and width of each inflorescence was determined using the best-fit ellipse (Figure 3A) in order to account for all variances or plasticity in its shape. The best fit ellipse is a least-squares approach based on central moments derived from image pixel positions of all the boundary pixels of a given binary object, where


$$\mathrm{object}\mathrm{length}=2\sqrt{\frac{2\left(u_{20}+u_{02}+\sqrt{{\left(u_{20}-u_{02}\right)}^{2}+4u_{11}^{2}}\right)}{u_{00}}},$$

$$\mathrm{object}\mathrm{width}=2\sqrt{\frac{2\left(u_{20}+u_{02}-\sqrt{{\left(u_{20}-u_{02}\right)}^{2}+4u_{11}^{2}}\right)}{u_{00}}},$$

$u_{pq}=\sum_{i=1}^{n}{\left(x_{i}-\bar{x}\right)}^{p}{\left(y_{i}-\bar{y}\right)}^{q}$ and (*x*_*i*_, *y*_*i*_) represent the image position of the *i*^th^ pixel in a boundary of *n* pixels.

The CH of each inflorescence was determined using Andrew’s monotone chain convex hull algorithm (Andrew, 1979). A geometric approach to determine the area of a typical polygon was performed using geometrics of the locations obtained from each set of CH pixels as shown in Supplementary Figure 2.

The estimated length of each object in terms of pixel number was converted into an absolute value *via* (*n*_l_*p_*l*_) where *n*_*l*_ is the length of an object in terms of pixel number and *p*_*l*_ is the length in centimeters of an image pixel. Likewise, the area of each object was converted to absolute values *via* $\left(n_{a}*p_{l}^{2}\right)$, where *n*_*a*_ represents the number of pixels making up the object and, given that the image pixels were square, then $p_{l}^{2}$ is the square of the length in centimeters of an image pixel. Based on these parameters, inflorescence shape (ISH) was calculated as the ratio between IW and IL and for each plant, the inflorescence number (IN) and total inflorescence coverage (TIC) were computed. Furthermore, for each plant, a composite image was constructed using its extracted inflorescence objects, sorted by surface size (Figure 2E).

### Data Standardization and Statistical Analysis

In the current study, comprehensive morphological attributes of 127,000 inflorescences have been recorded and analyzed across 478 cannabis plants. The recorded data contains representatives of both fresh (processed and imaged on harvest day, total of 209 plants) and dried (processed and imaged after drying, total of 283 plants) inflorescence material. To allow the integration between the two data sets, images of 14 plants were captured under both fresh and dry conditions. Based on these records, regression analysis between the 2 inflorescence states was performed for each parameter and a linear trendline and R^2^ values were generated to statistically evaluate the trendline prediction accuracy. Since all regressions have been characterized by R^2^ values greater than 0.9, the linear trendline equation was utilized to adjust “fresh” recorded data to its “dry” equivalent values (Supplementary Figure 3), in order to generate a unified and intact data set. In addition, it is important to note that plant cultivation methodologies which include practices such as plant pruning, vegetative growth phase duration, plant density etc., play a significant role in the determination of plant development and its inflorescence morphology. Thus, the presented absolute values reflect the growth conditions under which the current experiment took place.

Statistical analysis was performed by IBM SPSS Statistics for Windows, Version 26.0 (Armonk, NY: IBM Corp) and by R (R Core Team, 2020). For all examined inflorescences’ parameters, the mean and standard deviation (σ_*p*_) within plants were computed. Following this, phenotypic variation across clonal plant repetitions was assessed to evaluate environmental effects throughout the CE facility. Accordingly, spatial adjustments were applied to the data and an estimated value across replicates for each genotype was generated (as detailed in Naim-Feil et al., 2021). PCA (principal component analysis) and correlation matrix were generated using “corrplot” and “stats” R packages, respectively. ASReml models were used to calculate broad-sense heritability (H^2^) for all recorded parameters as the proportion of phenotypic variation (V_*P*_) that can be ascribed to genetic factors (V_*G*_) as H^2^ = V_*G*_/V_*P*_.

## Results

### Phenotypic Diversity

**FIGURE 4:**
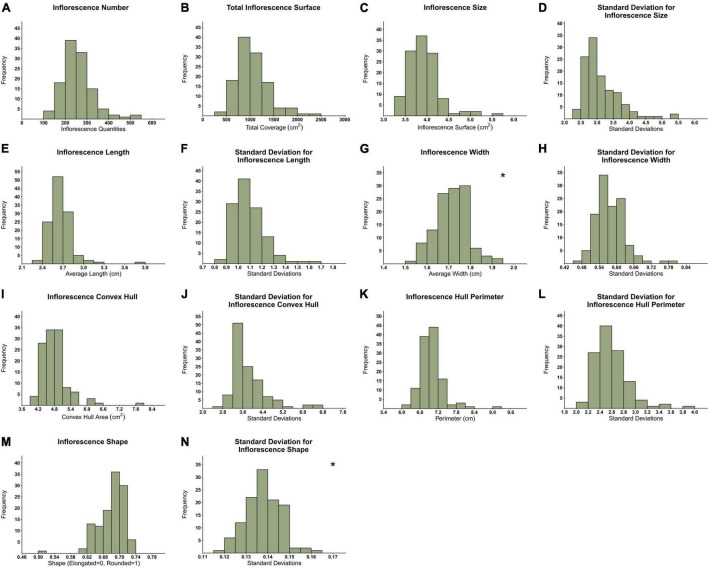
Frequency distribution of 119 genotypes across 14 inflorescences’ attributes. **(A)** Inflorescence Number (IN), **(B)** Total Inflorescence Surface Coverage (TIC), **(C)** Average Inflorescence Size (IS), **(D)** Inflorescence Size standard deviation (IS.sd), **(E)** Average Inflorescence Length (IL), **(F)** Inflorescence Length standard deviation (IL.sd), **(G)** Average Inflorescence Width (IW), **(H)** Inflorescence Width standard deviation (IW.sd), **(I)** Average Inflorescence Convex Hull (CH), **(J)** Inflorescence Convex Hull standard deviation (CH.sd), **(K)** Average Inflorescence Hull Perimeter (HP), **(L)** Inflorescence Hull Perimeter standard deviation (HP.sd), **(M)** Inflorescence Shape (ISH), **(N)** Inflorescence Shape standard deviation (ISH.sd). *Normally distributed figures marked with an asterisk on the top right corner.

The frequency distribution of the mean for all 119 examined genotypes (Figure 4) reflects the high variation in inflorescence morphology across all 14 recorded parameters. Although most presented histograms are not characterized by a normal distribution, in most cases, the removal of a few outlier genotypes can restore normality to the distribution. An examination of inflorescence quantity distribution (Figure 4A) reveals that while most genotypes are characterized by an average of 200–300 inflorescences per plant, some genotypes are typified by up to 500 per plant. Furthermore, the TIC of the examined genotypes ranges from 250 to 2,500 cm^2^ (Figure 4B). Hence, for some genotypes, the overall coverage of inflorescence surfaces can be up to 10 times greater than others. Following this, as depicted in Figure 4C, the average inflorescence size ranges from 3.25 to 5.5 cm^2^ across the examined plant population. Interestingly, a comparison between the distribution profile of IL (Figure 4E) and IW (Figure 4G) reveals that the latter is characterized by a narrower distribution range (0.45 cm) in comparison to the former (1.05 cm). Moreover, the standard deviation (sd) values of attributes such as IL, IW and ISH (Figures 4F,H,N, respectively) were found to be relatively narrow and low in comparison to the sd values of attributes such as CH and HP (Figures 4J,L, respectively).

### Broad–Sense Heritability (H^2^)

Broad-sense heritability estimations for inflorescence attributes are presented in Table 1. Across all indices concerning inflorescence attributes, ISH had the highest H^2^ value (0.38) while all other parameters had values below 0.3 with IS and CH generating the lowest H^2^ values of 0.15 and 0.16, respectively. Nevertheless, PH, DTM and IDB, physiological parameters which link to the plant performance as a whole unit, are characterized by relatively higher H^2^ values in comparison to most H^2^ entries recorded for inflorescences’ attributes (i.e., IW, TIC, HP).

**TABLE 1:** Broad-sense heritability (H^2^) for inflorescence morphological traits and key physiological attributes.

Trait	H^2^
Inflorescence Number	0.29
Inflorescence Size	0.15
Inflorescence Size Standard Deviation	0.18
Total Inflorescence Coverage	0.28
Inflorescence Length	0.18
Inflorescence Length Standard Deviation	0.17
Inflorescence Width	0.19
Inflorescence Width Standard Deviation	0.22
Convex Hull	0.16
Convex Hull Standard Deviation	0.18
Hull Perimeter	0.17
Hull Perimeter Standard Deviation	0.19
Inflorescence Shape	0.38
Inflorescence Shape Standard Deviation	0.22
Plant Height	0.52
Days to Maturation	0.49
Inflorescence Dry Biomass	0.33

### Traits Association

**FIGURE 5:**
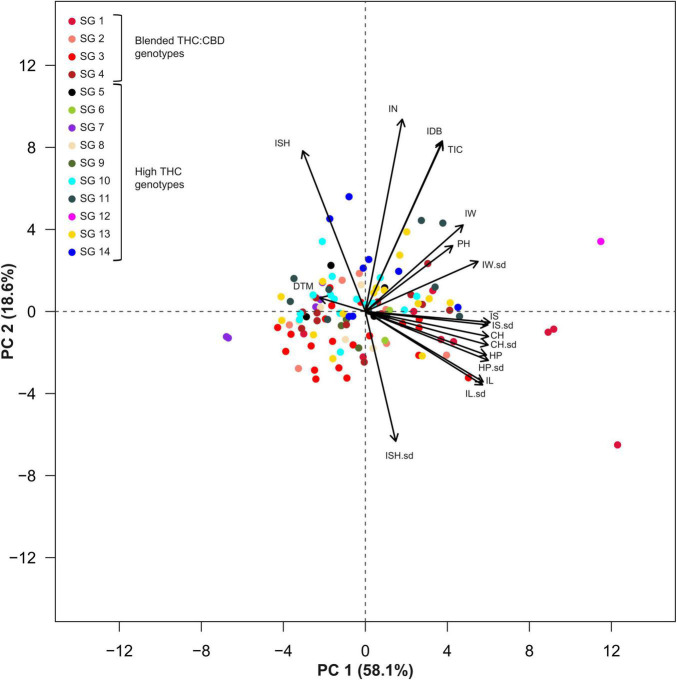
Principal Component Analysis (PCA) for 119 genotypes across 14 Inflorescences’ attributes and three physiological parameters. Colors indicate genotypic classifications into strain groups (SG) according to vernacular affiliations (for example, “Banana Kush” or “Diesel”). Genotypes marked in red (or a red shade) are characterized with a blended THC:CBD ratio while all other colors indicate high-THC genotypes (plants cannabinoid classification was assessed through DNA markers). Abbreviations: Inflorescence Size (IS), Inflorescence Size standard deviation (IS.sd), Convex Hull (CH), Convex Hull standard deviation (CH.sd), Hull Perimeter (HP), Hull Perimeter standard deviation (HP.sd), Inflorescence Length (IL), Inflorescence Length standard deviation (IL.sd), Inflorescence Width (IW), Inflorescence Width standard deviation (IW.sd), Total Inflorescence Coverage (TIC), Inflorescence Number (IN), Inflorescence Shape (ISH), Inflorescence Shape standard deviation (ISH.sd), Inflorescence Dry Biomass (IDB), Plant Height on harvest day (PH), Days To Maturation (DTM).

**FIGURE 6:**
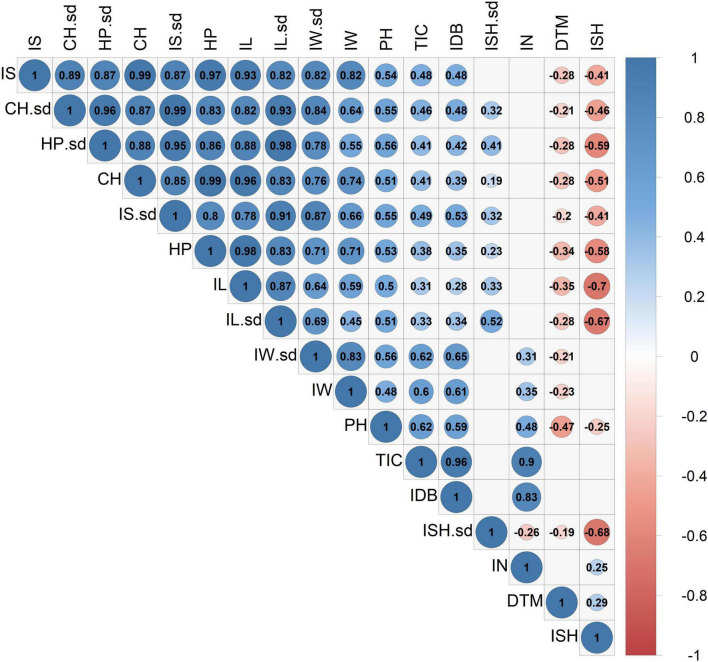
Correlation coefficient matrix for 14 inflorescences’ attributes and 3 physiological parameters. The correlation strength and direction are indicated by colors and numeric (coefficient) values. Blank cells indicate statistically insignificant correlations (*P* > 0.05). Abbreviations: Inflorescence Size (IS), Inflorescence Size standard deviation (IS.sd), Convex Hull (CH), Convex Hull standard deviation (CH.sd), Hull Perimeter (HP), Hull Perimeter standard deviation (HP.sd), Inflorescence Length (IL), Inflorescence Length standard deviation (IL.sd), Inflorescence Width (IW), Inflorescence Width standard deviation (IW.sd), Total Inflorescence Coverage (TIC), Inflorescence Number (IN), Inflorescence Shape (ISH), Inflorescence Shape standard deviation (ISH.sd), Inflorescence Dry Biomass (IDB), Plant Height on harvest day (PH), Days To Maturation (DTM).

A principal component analysis, as well as a correlation matrix, were performed (Figures 5, 6) in order to assess traits association across all recorded inflorescence parameters and key physiological traits. Although the two main components presented in Figure 5, jointly explain 76.7% of the variation (PC1 and PC2 accounting for 58.1 and 18.6%, respectively), a highly correlated vectors aggregate, containing parameters that are associated with the dimensions of the intact inflorescence (CH, HP, IS), was observed over the 4th quadrant of the Cartesian system. Interestingly, this group also includes the IL parameter while it does not comprise the IW vector that appears to be highly associated with the plant yield (IDB). The close association between IDB and the average IW is also corroborated through the correlation matrix (Figure 6) with a relatively high coefficient value (*r* = 0.61). In addition, each of the inflorescence morphological attributes that comprise this cluster was found to be highly associated with its sd parameter (Figure 5) that is characterized by a statistically significant (*p* < 0.05) correlation coefficient value of *r* = 0.87, *r* = 0.86, *r* = 0.87 and *r* = 0.87 for CH, HP, IS and IL, respectively (Figure 6).

From the correlation matrix (Figure 6), IDB demonstrates a stronger association with IN (*r* = 0.83) than with IS (*r* = 0.48). Yet, IS was found to be correlated with TIC (*r* = 0.48) but no correlation was demonstrated between IS and IN. Plants’ precocity (low DTM) was found to be correlated with elongated (DTM to IL, *r* = −0.35), wider (DTM to IW, *r* = −0.23) and larger inflorescences (IS, *r* = −0.28) and in contrast, late maturation (high DTM) was found to be correlated with rounded inflorescences (DTM to ISH, *r* = 0.29) while no correlation was detected between a specific ISH and IDB.

### The Effect of Inflorescence Size on the Overall Yield

**FIGURE 7:**
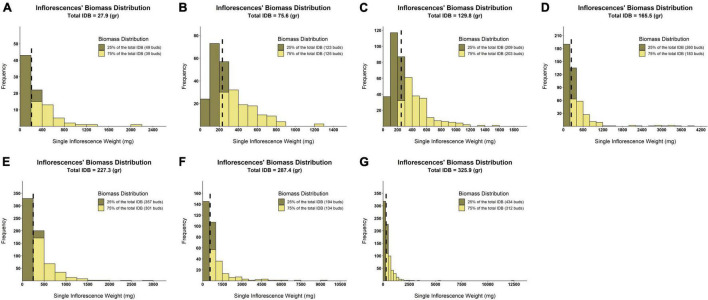
Distribution histograms of within-plant inflorescences’ weight across various levels of IDB. Surfaces colored in khaki represent the quantity of smaller inflorescences that contributes to the accumulation of 25% of plant IDB. Surfaces colored in yellow reflect the quantity of larger inflorescences that contributes to the accumulation of 75% of plant IDB. Black broken lines indicate the inflorescences’ weight median for each of the examined plants. **(A)** IDB = 27.9 (gr/plant), **(B)** IDB = 75.6 (gr/plant), **(C)** IDB = 129.8 (gr/plant), **(D)** IDB = 165.5 (gr/plant), **(E)** IDB = 227.3 (gr/plant), **(F)** IDB = 287.4 (gr/plant), and **(G)** IDB = 325.9 (gr/plant).

From the principal component analysis (Figure 5), despite IDB and TIC being independently recorded parameters, their vectors are completely overlapping. This association is reinforced by the high correlation coefficient value (*r* = 0.96, Figure 6). Accordingly, a unique ratio of gram per cm^2^ was calculated for each plant by dividing the plant’s IDB (gr) by its TIC (cm^2^). The obtained ratio was then utilized to compute the weight of each inflorescence, in order to assess the profile of inflorescence weight distribution across the examined plants as demonstrated in Figure 7, from 7 selected plants, chosen to represent the IDB spectrum (range from 27.9 to 325.9 gr/plant). As depicted in Figure 7, the smallest inflorescences (weighing less than 200 mg) are highly prevalent but their contribution to the overall weight is limited. In addition, it was found that across most plants, the larger inflorescences, accounting for less than 50% of the overall inflorescence quantity, contributes to 75% or more of the plant’s total IDB.

**FIGURE 8:**
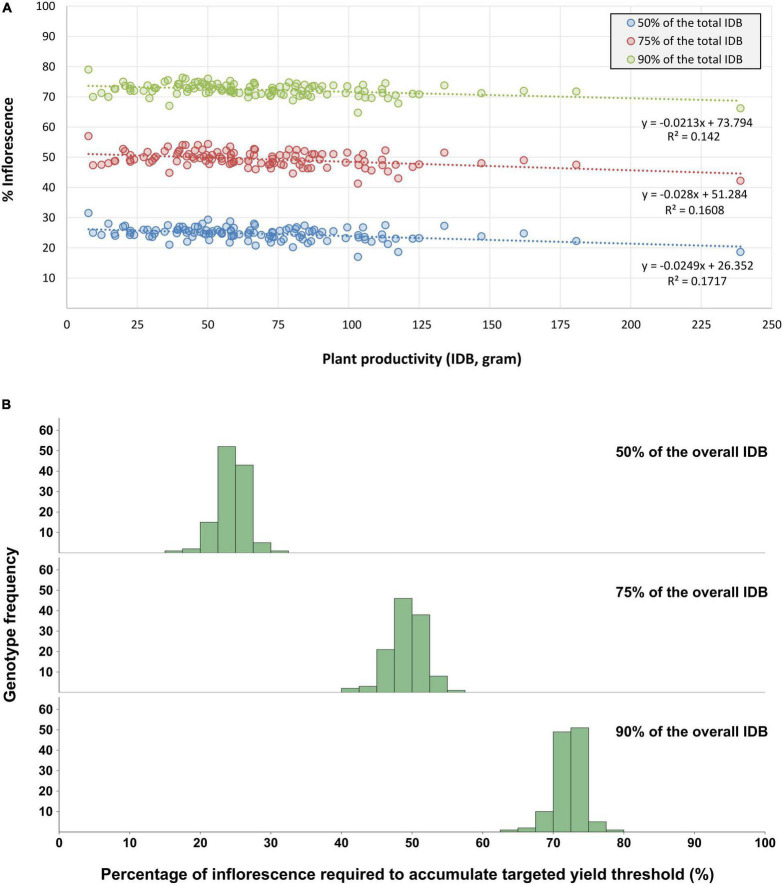
Percentage of inflorescences required for the accumulation of 50%, 75% and 90% of the overall IDB. The presented data refers to the proportion between the larger inflorescences quantity (required to accumulate the targeted yield threshold) and the overall inflorescences quantity within each plant. **(A)** Illustrates the percentage dispersal across all genotypes for each of the targeted yield thresholds. Linear regression trendlines and their corresponding datapoints are marked with the same color, **(B)** Presents the frequency distribution of all examined genotypes across the three targeted yield thresholds.

Furthermore, the overall percentage of inflorescences that need to be extracted and processed, in order to accumulate 50, 75, and 90% of the plant’s IDB was calculated and ranged between 17–32%, 41–58% and 64–79% of the total inflorescence quantity, respectively (Figure 8). The trendline equations obtained by linear regressions between inflorescence quantity and the targeted yield thresholds (to accumulate 50, 75, and 90% of the overall IDB) across all genotypes are characterized by statistically significant (*P* < 0.001) coefficients values (slope) of *r* = −0.025, *r* = −0.028, *r* = −0.021, and *R*^2^ values of 0.17, 0.16 and 0.14, respectively.

### Yield Assessment and Prediction Equation

Due to the relatively high correlation coefficient value observed between IW and IDB (*r* = 0.61, Figure 6), a linear regression analysis was performed to predict plant productivity *via* IW measurements. To be relevant in a production environment with plants nearing harvest under cultivation conditions, the regression model was run using estimated values of fresh rather than dried IW. In addition, as cannabis plant productivity is often expressed by copious inflorescence quantities (as demonstrated through Figures 6, 7), which are challenging to measure under field conditions, a limited number of inflorescences were selected and defined in order to generate a yield assessment methodology. Based on the assumption that the plant’s longer inflorescences form over the branches’ apex, the top 20 longer inflorescences within each plant were examined and their corresponding width data was extracted and averaged. Furthermore, to improve the prediction accuracy by focusing on a feasible IDB range desired for selection purposes (above∼ 15 gr/plant), the regression model was performed using plants with an IW average greater than 2.5 cm (*n* = 426). Prior to running the regression analysis, a linear relationship was observed between the examined variables for the prediction model and assumptions of homoscedasticity and normality of the residuals were met. IW predicted the plant’s IDB, *F*(1, 424) = 549.77, *p* < 0.001, accounting for 56% of the variation in IDB (*R*^2^ = 0.56). Thus, the following prediction equation for IDB was generated:


$$\;{\text{IDB}}_{\left(gr/plant\right)}=59.14\times IW_{\left(cm\right)}^{*}-134.97$$

Refers to the average IW of the longest 20 inflorescences.*

**FIGURE 9:**
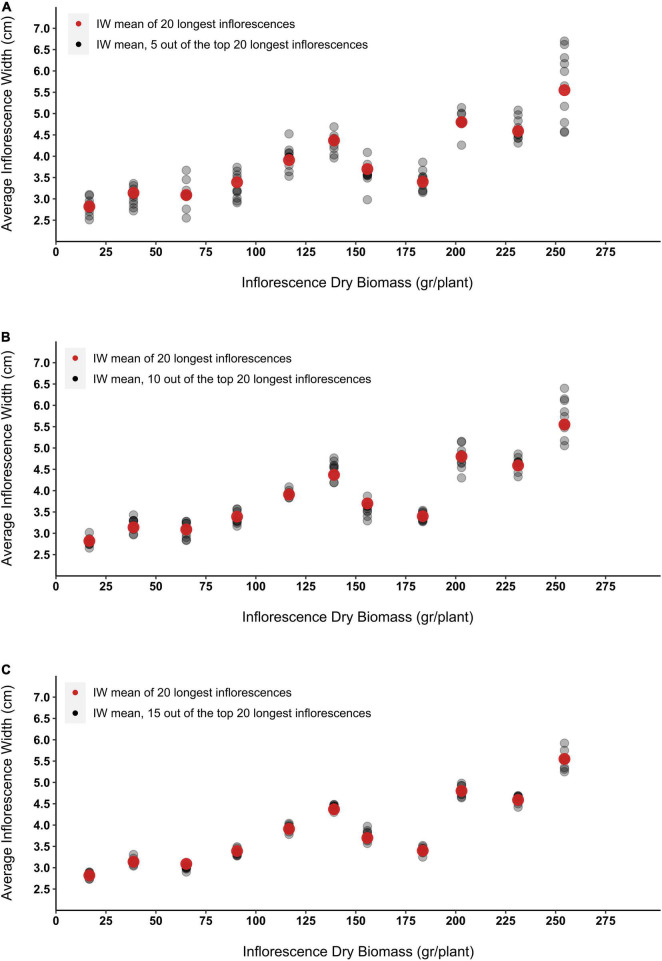
The effect of inflorescence width (IW) measurement sample size on the accuracy of the obtained mean data. Red dots represent the mean of the entire sample group (the width of the top 20 longest inflorescences). Grey dots represent the inflorescence width average that was obtained from 10 sampling rounds of **(A)** 5 randomly selected inflorescences (out of the top 20 longest inflorescences), **(B)** 10 randomly selected inflorescences (out of the top 20 longest inflorescences), and **(C)** 15 randomly selected inflorescences (out of the top 20 longest inflorescences).

To simulate errors that may occur while visually evaluating the longest 20 epical inflorescences emerging on a growing plant, the mean IW of 5, 10 and 15 inflorescences that were randomly selected out of the longest 20 inflorescences was calculated. For each of the examined case groups, 10 sample rounds were applied and the dispersal of the obtained averages around the absolute width mean of the 20 longer inflorescences was assessed (Figure 9). Although with more inflorescences selected, the overall variance of the predicted average IW decreases, across all examined IDB range, the presented simulation demonstrates moderate to low variation in IW for sample groups of 10 and 15 inflorescences (Figures 9B,C). For example, IDB prediction accuracy for the most productive plant presented in Figure 9 (IDB = 254 gr) reveals that while the absolute IW average of the longest 20 inflorescences of this plant measured as 5.55 cm, the divergent from the actual mean is 20% in size sample of 5 (range between 4.56–6.7 cm), 15% in size sample of 10 (range between 5.05–6.4 cm) and 7% in size sample of 15 (range between 5.25–5.92 cm).

## Discussion

The current study focuses on the role of various cannabis inflorescence attributes in the determination of the overall plant’s yield component and their association with key physiological and phenological traits. The examined germplasm includes 119 cannabis drug-type genotypes, which were obtained from legal medicinal cannabis commercial companies, and comprise 74 genotypes characterized by a high-THC:low-CBD ratio and 45 genotypes with a blended THC:CBD ratio. Although cannabis recreational breeding has reportedly impacted the cannabinoids’ genetic diversity by intensifying and selecting high THC genotypes (Hazekamp, 2007; Mehmedic et al., 2010; Cascini et al., 2012; Chouvy, 2019), the current examination of inflorescence morphological attributes reveals a broad genotypic diversity across all examined parameters (Figure 4). These findings are aligned with the previously reported diversity that was identified across physiological and phenological attributes of the current medicinal cannabis germplasm (Naim-Feil et al., 2021). Nevertheless, to broaden this conclusion for genotypes characterized by low THC:high CBD cannabinoid profile, further studies are required.

### Heritability

The broad-sense heritability (H^2^) estimations presented in this study can be divided into 2 cohorts: (1) Indices associated with attributes of the intact plant (e.g., PH, TIC, IN) and (2) Indices that focus on features of processed inflorescences (e.g., IW, ISH, CH). As depicted in Table 1, all H^2^ entries recorded for parameters within the first cohort are greater than the estimated values recorded for the second cohort (excluding ISH). These findings suggest that under the current experiment, inflorescence features are more susceptible to environmental factors as compared to physiological and phenological traits of the intact plant. Although studies examining the heritability indices of cannabis inflorescence features are scarce, a similar H^2^ value to that of cannabis IS (0.15) was found among the size of safflower heads (0.21, Camas and Esendal, 2006). In addition, Gómez et al. (2009) investigated the narrow-sense heritability (h^2^) of *Erysimum mediohispanicum* (Brassicaceae) flower features and reported an h^2^ value of 0.24 for flower size, 0.26 for flower length (in comparison to IL H^2^ of 0.18 in the current study) and 0.019 for flower width (in comparison to IW H^2^ of 0.19 in the current study). Although these heritability estimations were examined across plants of different taxonomic orders, they all indicate that environmental factors play a major role in the determination of the flowers’ phenotypic profile. Therefore, with the relatively low H^2^ values typifying the characteristics of cannabis inflorescences, it is anticipated that these traits will have a limited response to selection. Hence, it is suggested that the production of inflorescences with desired morphology will combine breeding initiatives, studies examining the genotype X environment interactions and rigorous adjustments of environmental factors within the cultivation facility.

### Traits Association

The close association between traits related to the inflorescence magnitude (e.g., IS, IL, HP) and their compatible standard deviation (sd) parameter (Figures 5, 6) indicates that as the average inflorescence size increases, so does the variation across the inflorescences’ size within the examined plant (increased sd). However, the significant role of environmental factors in the determination of inflorescences morphology (as depicted in Table 1) and the range of different microenvironmental conditions to which different areas of the plant are exposed suggest that through the implementation of adjusted cultivation methodologies such as: tailored light intensity (Rodriguez-Morrison et al., 2021) and light spectra (Reichel et al., 2021), supplemented subcanopy light (Hawley et al., 2018), pruning (Small, 2017b; Gaudreau et al., 2020) and applications of exogenous growth regulators (Burgel et al., 2020), the variability across intra-plant inflorescence characteristics might be restrained.

In addition, the association between ISH and ISH-sd (Figure 6, *r* = −0.68) indicates that rounded inflorescences (ISH closer to 1) are likely to be consistent across the plant (low ISH-sd) while a varied shape morphology (high ISH-sd) is anticipated within genotypes characterized by elongated inflorescences (ISH closer to 0). Together with the relatively high H^2^ value that typifies ISH (Table 1, 0.38), these findings suggest that reproduction of rounded inflorescences as a commercial product can be relatively feasible to perform. Nevertheless, it should be noted that due to the correlation between ISH and DTM (Figure 6, *r* = 0.29), breeding for this trait might come at a cost of late-maturing phenology. In contrast to this, the association between early maturing and elongated (DTM to IL, *r* = −0.35), wider (DTM to IW, *r* = −0.23) and therefore larger inflorescences (DTM to IS, *r* = −0.28) indicate that selection for precocious genotypes is likely to be coupled with inflorescence enlargement. yet, no significant correlation was found between precocity (DTM) and productivity (IDB).

IDB displays a greater correlation coefficient value with IN than with IS (Figure 6, *r* = 0.83 and 0.48, respectively). Hence, for breeding initiatives striving to improve plant productivity by focusing on inflorescences’ attributes, selection for high inflorescence quantity will provide greater advances on the overall yield enhancement in comparison to selection for increased individual inflorescence sizes.

In the current study, both IL and IW demonstrate significant correlations with IDB (Figure 6, *r* = 0.28 and *r* = 0.61, respectively). However, the correlation coefficients values generated indicate that IW can be used as a better predictor for plant productivity than IL. Interestingly, although both IW and IL determine the ISH parameter, no significant association was found between IDB and ISH (Figure 6), which suggests that inflorescence profiles across prolific genotypes may not be limited to specific shapes. This is not surprising as the phenomenon of weak associations between the overall plant productivity and the shape of the commercial products (rounded vs. elongated) have been previously reported for several crops such as wheat (Gegas et al., 2010; Yoshioka et al., 2019) and tomatoes (Tanksley, 2004). More specifically, these studies indicated that the independence of these parameters was due to the presence of several loci that regulate fruit shape and other loci which regulate fruit size. However, this is the first study, to the authors’ knowledge to demonstrate this effect in cannabis inflorescence.

In the current study, inflorescence parameters within each plant were extracted from the analysis of a single image providing a contour-based two-dimensional (2D) assessment for each object. The high correlation found between TIC and IDB (Figure 6, *r* = 0.96) suggests that 2D measurements can be used for the assessment of inflorescence weight without the need for more complex volumetric (3D) measurements. This finding can be used to facilitate the characterization of cannabis plants for selection and breeding purposes as well as identifying optimal environmental conditions for enhancing crop productivity by applying a relatively simple measurement approach. This corresponds with the observations of Diago et al. (2015) in their examination of 2D vs. 3D image analysis as a tool for predicting yield weight in grapevine, where they concluded that 2D measurements can be used as a simple alternative to volumetric measurements when the examined objects are characterized by symmetrical shape.

### The Association Between Intra-Plant Inflorescence Weight Distribution and the Overall Plant Productivity

Although cannabis post-harvest operations are labor intensive (Carpentier et al., 2012), formal studies that estimate the expenses of these activities are scarce as their costs appear to vary extensively (Caulkins, 2010). With regards to Inflorescence weight distribution and its association with the overall IDB (Figure 7), our findings suggest that a cost-benefit assessment for extracting and processing the smaller inflorescences should be performed in order to optimize labor input per processed IDB unit. Interestingly, for nearly all of the examined plants, extracting the largest 50% of inflorescences led to a gain of 75% or more of the overall IDB. Furthermore, according to Figure 7, processing inflorescences weighing less than 200 mg has a relatively minor contribution to the final harvested weight.

This conclusion is underpinned also by the association between yield accumulation and the percentage of processed inflorescences (Figure 8) when harvesting 50, 75, and 90% of the total IDB of a given plant, it is required to process (approximately) its larger 25, 50, and 75% inflorescences, respectively. Thus, these findings may indicate diminishing returns to IDB for processing the 25% smaller inflorescences as these are contributing to only 10% of the overall yield. Moreover, the association between inflorescences’ proportion and gaining a comparable yield percentage was found to be consistent within each of the examined yield thresholds and across all IDB spectrum (Figure 8). Therefore, it is suggested that targeting desired yield threshold by processing a selected number of inflorescences can optimize cannabis production cost-benefit efficacy across a vast range of cultivated genotypes. Nevertheless, as smaller inflorescences typically form within the plant’s foliage where environmental factors such as light, humidity and aerate are suboptimal, it is expected that smaller and larger inflorescences will contain different cannabinoid compositions (Bernstein et al., 2019b). Therefore, a chemotypic characterization should be performed for each of the targeted yield thresholds, in order to evaluate the precise cannabinoid content within the processed plant material.

### Prediction Equation

Currently, the knowledge that can facilitate precision breeding for generating scientifically based cannabis strains is limited (Chouvy, 2019; Challa et al., 2020). Therefore, practical tools that enable accelerated plant screening and improve selection accuracy are valuable for the growing medicinal cannabis industry. However, unlike classical breeding programs aiming for the production of inbred varieties or hybrid seeds (F1) through crosses of genetically-stabilized parental lines, in cannabis, due to its typical vegetative propagation technique (Barcaccia et al., 2020), any hybrid plant in the observation facility, regardless of its parental genetic background, can result in a commercial variety. Thus, increasing the number of screened individuals directly enhances the probability to locate desirable varieties, but at the same time requires a tedious, costly and highly time-consuming phenotypic evaluation (e.g., cannabinoids and terpenoids content, phenology and physiological traits) that first and foremost includes yield assessments. The prediction equation presented in this study will enable estimates of plant productivity (IDB) by measuring the average width of the plant’s largest inflorescences which typically evolve on the branches apex (Dach et al., 2015; Reichel et al., 2021). Previously, a minimum number of 20 second-order branches per plant in the current plant population was identified (Naim-Feil et al., 2021). Thus, the proposed prediction equation was generated accordingly and requires the average width of 10 out of the top 20 randomly selected apical inflorescences of a given plant. Per the prediction equation, an increase of 1 cm in the calculated IW, will enhance the overall IDB by 59.14 gr. By using an on-site simple caliper measurement, this method allows for rapid yield forecasting estimation of diverse drug-type cannabis plant populations. However, as observed by Dach et al. (2015) and confirmed by the current study findings, environmental factors can have an extensive effect on cannabis inflorescence morphology which includes IW. Thus, the accuracy of the proposed prediction equation is expected to vary under different environmental conditions. To address this issue, one possibility is to forecast the IDB ratio between plants of a given population according to their compatible IW ratios. The linear association and the relatively high correlation coefficient between IDB and IW provide support for this method. Although this alternative approach does not provide an absolute prediction value for the plant’s IDB, it can be used as a practical tool for yield estimation under diverse environmental conditions and for heterogeneous plant populations. However, as this methodology has not yet been rigorously explored, its accuracy should be evaluated and validated by future studies.

## Conclusion

The findings presented in the current study indicate that environmental factors play a major role in the determination of inflorescences morphology. Therefore, it is suggested that the production of inflorescences with desired features will combine breeding activities, research examining the genotype X environment interactions and rigorous adjustments of environmental factors within the cultivation facility. With regards to inflorescence weight distribution, our findings suggest that processing 75% of the plant’s largest inflorescence will gain 90% of the plant’s yield potential. Therefore, it is worthwhile to evaluate if the benefits from extracting and processing the plant’s 25% smaller inflorescences outweigh its operational costs. Based on the relatively high correlation between plant productivity (IDB) and inflorescence width (IW), a prediction equation for forecasting the plant’s IDB through width measurements of specific inflorescences was generated. However, since this equation was generated based on inflorescences’ width that formed under specific cultivation settings, to expand this selection methodology for diverse environmental conditions it is proposed to rate the predicted IDB values according to the IW ratios within populations cultivated under the same growth conditions.

The knowledge obtained in the current study can facilitate the generation of desired inflorescences, improve yield productivity and increase labor efficacy in commercial production pipelines. To build on this work, future studies could investigate inflorescences’ features on a microscopic level to further explore trichomes morphology and density. Moreover, further research is needed to investigate the genetic factors that regulate inflorescence morphology, plant physiology and cannabinoids biosynthesis. These, together with the insights of this research, will improve our capability to generate and cultivate scientifically based cannabis cultivars for medicinal application.

## Data Availability Statement

The original contributions presented in the study are included in the article/Supplementary Material, further inquiries can be directed to the corresponding author/s.

## Author contributions

EN-F designed the study, performed the statistical analysis, and prepared the manuscript. EN-F and LS cultivated and processed the plant material, captured, scaled, and manually refined all images. EB designed and programmed the image analysis software and extracted inflorescence evaluation data. LP performed the trial’s design and implemented the spatial adjustments. NC and GS co-supervised all aspects of the project and assisted with the manuscript preparation and secured funding. All authors have approved the manuscript.

## Conflict of Interest

The authors declare that the research was conducted in the absence of any commercial or financial relationships that could be construed as a potential conflict of interest.

## Publisher’s Note

All claims expressed in this article are solely those of the authors and do not necessarily represent those of their affiliated organizations, or those of the publisher, the editors and the reviewers. Any product that may be evaluated in this article, or claim that may be made by its manufacturer, is not guaranteed or endorsed by the publisher.

## Abbreviations

IN: Inflorescence Number
TIC: Total Inflorescence Coverage
IS: Inflorescence Size
IS-sd: Inflorescence Size standard deviation
IL: Inflorescence Length
IL-sd: Inflorescence Length standard deviation
IW: Inflorescence Width
IW-sd: Inflorescence Width standard deviation
CH: Convex Hull
CH-sd: Convex Hull standard deviation
HP: Hull perimeter
HP-sd: Hull perimeter standard deviation
ISH: Inflorescence Shape
ISH-sd: Inflorescence Shape standard deviation
IDB: Inflorescence Dry Biomass
PH: Plant Height on harvest day
DTM: Days To Maturation
CE: Controlled Environment
RH: Relative Humidity.

## References

[B1] Abel E. L. (1980). *Marihuana the First Twelve Thousand Years.* Berlin: Springer Science+Business Media.

[B2] Andre C. M. Hausman J. F. Guerriero G. (2016). Cannabis sativa: the plant of the thousand and one molecules. *Front. Plant Sci.* 7:19. 10.3389/fpls.2016.00019 26870049 PMC4740396

[B3] Andrew A. M. (1979). Another efficient algorithm for convex hulls in two dimensions. *Inf. Process. Lett.* 9 216–219. 10.1016/0020-0190(79)90072-90073

[B4] Barcaccia G. Palumbo F. Scariolo F. Vannozzi A. Borin M. Bona S. (2020). Potentials and challenges of genomics for breeding cannabis cultivars. *Front. Plant Sci.* 11:573299. 10.3389/fpls.2020.573299 33101342 PMC7546024

[B5] Baron E. P. (2018). Medicinal properties of cannabinoids, terpenes, and flavonoids in cannabis, and benefits in migraine, headache, and pain: an update on current evidence and cannabis science. *Curr. Pain Headache Rep.* 58 1139–1186. 10.1111/head.13345 30152161

[B6] Bernstein N. Gorelick J. Koch S. (2019a). Interplay between chemistry and morphology in medical cannabis (*Cannabis sativa* L.). *Ind. Crops Prod.* 129 185–194. 10.1016/j.indcrop.2018.11.039

[B7] Bernstein N. Gorelick J. Zerahia R. Koch S. (2019b). Impact of N, P, K, and humic acid supplementation on the chemical profile of medical cannabis (*Cannabis sativa* L). *Front. Plant Sci.* 10:736. 10.3389/fpls.2019.00736 31263470 PMC6589925

[B8] Breen E. J. Jones R. (1996). Attribute openings, thinnings, and granulometries. *Comput. Vis. Image Underst.* 64 377–389. 10.1006/cviu.1996.0066

[B9] Burgel L. Hartung J. Schibano D. (2020). Impact of different phytohormones on morphology, yield and cannabinoid content of *Cannabis sativa* L. *Plants* 9:725. 10.3390/plants9060725 32521804 PMC7355821

[B10] Cai J. Kumar P. Chopin J. Miklavcic S. J. (2018). Land-based crop phenotyping by image analysis: accurate estimation of canopy height distributions using stereo images. *PLoS One* 13:e0196671. 10.1371/journal.pone.0196671 29795568 PMC5967702

[B11] Cai J. Zeng Z. Connor J. N. Huang C. Y. Melino V. Kumar P. (2015). RootGraph: a graphic optimization tool for automated image analysis of plant roots. *J. Exp. Bot.* 66 6551–6562. 10.1093/jxb/erv359 26224880 PMC4623675

[B12] Camas N. Esendal E. (2006). Estimates of broad-sense heritability for seed yield and yield components of grass pea (*Lathyrus sativus* L.). *Hereditas* 143 55–57. 10.3906/tar-0611-622 17362334

[B13] Caplan D. Dixon M. Zheng Y. (2017). Optimal rate of organic fertilizer during the flowering stage for cannabis grown in two coir-based substrates. *HortScience* 52 1796–1803. 10.21273/HORTSCI12401-17

[B14] Caplan D. Dixon M. Zheng Y. (2019). Increasing inflorescence dry weight and cannabinoid content in medical cannabis using controlled drought stress. *HortScience* 54 964–969. 10.21273/HORTSCI13510-18

[B15] Carpentier C. Mulligan K. Laniel L. Potter D. Hughes B. Vandam L. (2012). *Cannabis Production and Markets in Europe.* Lisbon: EMCDDA.

[B16] Cascini F. Aiello C. Di Tanna G. (2012). Increasing Delta-9-Tetrahydrocannabinol (Delta -9-THC) content in herbal cannabis over time: systematic review and meta-analysis. *Curr. Drug Abuse Rev.* 5 32–40. 22150622 10.2174/1874473711205010032

[B17] Cascio M. G. Pertwee R. G. Marini P. (2017). “The pharmacology and therapeutic potential of plant cannabinoids,” in *Cannabis sativa L. - Botany and Biotechnology*, eds Chandra S. Lata H. ElSohly M. A. (Berlin: Springer International Publishing), 207–225. 10.1007/978-3-319-54564-6_9

[B18] Caulkins J. P. (2010). *Estimated Cost of Production for Legalized Cannabis.* Santa Monica, CA: Rand.

[B19] Challa S. K. R. Misra N. N. Martynenko A. (2020). Drying of cannabis—state of the practices and future needs. *Dry. Technol.* 39 2055–2064. 10.1080/07373937.2020.1752230

[B20] Chandra S. Lata H. ElSohly M. A. (2020). Propagation of cannabis for clinical research: an approach towards a modern herbal medicinal products development. *Front. Plant Sci.* 11:58. 10.3389/fpls.2020.00958 32676092 PMC7333344

[B21] Chouvy P.-A. (2019). Cannabis cultivation in the world: heritages, trends and challenges. *EchoGéo* 48 1–20. 10.4000/echogeo.17591

[B22] Clarke R. Merlin M. (2013). *Cannabis- Evolution and Ethanobotany.* Los Angeles, CA: University of California Press, 771–810.

[B23] Dach J. Moore E. A. Kander J. (2015). “Cannabisbotany, taxonomy and growth,” in *Cannabis Extracts in Medicine The Promise of Benefits in Seizure Disorders, Cancer and Other Conditions*, (Jefferson NC: McFarland & Company, Inc., Publishers), 21–36.

[B24] Danziger N. Bernstein N. (2021a). Light matters: effect of light spectra on cannabinoid profile and plant development of medical cannabis (*Cannabis sativa* L.). *Ind. Crops Prod.* 164:113351. 10.1016/j.indcrop.2021.113351

[B25] Danziger N. Bernstein N. (2021b). Plant architecture manipulation increases cannabinoid standardization in ‘drug-type’ medical cannabis. *Ind. Crops Prod.* 167:113528. 10.1016/j.indcrop.2021.113528

[B26] Dayanandan P. Kaufman P. B. (1976). Trichomes of *Cannabis sativa* L. (Cannabaceae). *Am. J. Bot.* 63 578–591. 10.1002/j.1537-2197.1976.tb11846.x

[B27] Diago M. P. Tardaguila J. Aleixos N. Millan B. Prats-Montalban J. M. Cubero S. (2015). Assessment of cluster yield components by image analysis. *J. Sci. Food Agric.* 95 1274–1282. 10.1002/jsfa.6819 25041796

[B28] Ebersbach P. Stehle F. Kayser O. Freier E. (2018). Chemical fingerprinting of single glandular trichomes of Cannabis sativa by Coherent anti-Stokes Raman scattering (CARS) microscopy. *BMC Plant Biol.* 18:275. 10.1186/s12870-018-1481-1484 30419820 PMC6233497

[B29] ElSohly M. A. Radwan M. M. Gul W. Chandra S. Galal A. (2017). “Phytochemistry of *Cannabis sativa* L,” in *Phytocannabinoids. Progress in the Chemistry of Organic Natural Products*, eds Kinghorn A. D. Falk H. Gibbons S. Kobayashi J. (Cham: Springer International Publishing), 1–36. 10.1007/978-3-319-45541-9_1 28120229

[B30] Erkelens J. L. Hazekamp A. (2014). An essay on the history of the term Indica and the taxonomical conflict between the monotypic and polytypic views of Cannabis. *Cannabinoids* 9 9–15.

[B31] Flores-sanchez I. J. Verpoorte R. (2008). Secondary metabolism in cannabis. *Phytochem. Rev.* 7 615–639. 10.1007/s11101-008-9094-9094

[B32] Gage J. L. Miller N. D. Spalding E. P. Kaeppler S. M. de Leon N. (2017). TIPS: a system for automated image-based phenotyping of maize tassels. *Plant Methods* 13:21. 10.1186/s13007-017-0172-178 28373892 PMC5374692

[B33] Gaudreau S. Missihoun T. Germain H. (2020). Early topping: an alternative to standard topping increases yield in cannabis production. *Plant Sci. Today* 7 627–630. 10.14719/PST.2020.7.4.927

[B34] Gegas V. C. Nazari A. Griffiths S. Simmonds J. Fish L. Orford S. (2010). A genetic framework for grain size and shape variation in wheat. *Plant Cell* 22 1046–1056. 10.1105/tpc.110.074153 20363770 PMC2879751

[B35] Godfray H. C. J. Beddington J. R. Crute I. R. Haddad L. Lawrence D. Muir J. F. (2010). Food security: the challenge of Feeding 9 Billion People. *Science* 327 812–818. 10.1016/j.geoforum.2018.02.030 20110467

[B36] Goldstein B. B. (2015). *Cannabis in the Treatment of Pediatric Epilepsy.* Chicago, IL: O’Shaughnessy’s.

[B37] Gómez J. M. Abdelaziz M. Muñoz-Pajares J. Perfectti F. (2009). Heritability and genetic correlation of corolla shape and size in erysimum mediohispanicum. *Evolution* 63 1820–1831. 10.1111/j.1558-5646.2009.00667.x 19245399

[B38] Hall W. Degenhardt L. (2007). Prevalence and correlates of cannabis use in developed and developing countries. *Curr. Opin. Psychiatry* 20 393–397. 10.1097/YCO.0b013e32812144cc 17551355

[B39] Hammond C. T. Mahlberg P. G. (1973). Morphology of glandular hairs of cannabis sativa from scanning electron microscopy. *Am. J. Bot.* 60 524–528. 10.1002/j.1537-2197.1973.tb05953.x

[B40] Hammond C. T. Mahlberg P. G. (1977). Morphogenesis of capitate glandular hairs of *Cannabis sativa* (Cannabaceae). *Am. J. Bot.* 64 1023–1031. 10.1002/j.1537-2197.1977.tb11948.x

[B41] Hand A. Blake A. Kerrigan P. Samuel P. Friedberg J. (2016). History of medical cannabis. *J. Pain Manag.* 9 387–394.

[B42] Hawley D. Graham T. Stasiak M. Dixon M. (2018). Improving Cannabis bud quality and yield with subcanopy lighting. *HortScience* 53 1593–1599. 10.21273/HORTSCI13173-18

[B43] Hazekamp A. (2007). “An evaluation of the quality of medicinal grade cannabis in the Netherlands,” in *Cannabis; Extracting the Medicine*, ed. Ipskamp B. V. (Amsterdam: PrintPartners), 25–38.

[B44] Jahnke S. Roussel J. Hombach T. Kochs J. Fischbach A. Huber G. (2016). phenoSeeder - a robot system for automated handling and phenotyping of individual seeds. *Plant Physiol.* 172 1358–1370. 10.1104/pp.16.01122 27663410 PMC5100762

[B45] Jeudy C. Adrian M. Baussard C. Bernard C. Bernaud E. Bourion V. (2016). RhizoTubes as a new tool for high throughput imaging of plant root development and architecture: test, comparison with pot grown plants and validation. *Plant Methods* 12:31. 10.1186/s13007-016-0131-139 27279895 PMC4897935

[B46] Jin D. Dai K. Xie Z. Chen J. (2020). Secondary metabolites profiled in cannabis inflorescences, leaves, stem barks, and roots for medicinal purposes. *Sci. Rep.* 10:3309. 10.1038/s41598-020-60172-60176 32094454 PMC7039888

[B47] Kipp S. Mistele B. Baresel P. Schmidhalter U. (2014). High-throughput phenotyping early plant vigour of winter wheat. *Eur. J. Agron.* 52 271–278. 10.1016/j.eja.2013.08.009

[B48] Kittler J. Illingworth J. (1986). Minimum error thresholding. *Pattern Recognit.* 19 41–47. 10.1016/0031-3203(86)90030-90030

[B49] Lata H. Chandra S. Uchendu E. E. Khan A. I. ElSohly M. A. (2019). “Cultivating research grade cannabis for the development of phytopharmaceuticals,” in *Medicinal Plants From Farm to Pharmacy*, eds Joshee Dhekney Parajuli (Cham: Springer Nature) 169–186.

[B50] Leweke F. M. Piomelli D. Pahlisch F. Muhl D. Gerth C. W. Hoyer C. (2012). Cannabidiol enhances anandamide signaling and alleviates psychotic symptoms of schizophrenia. *Transl. Psychiatry* 2:e94. 10.1038/tp.2012.15 22832859 PMC3316151

[B51] Livingston S. J. Quilichini T. D. Booth J. K. Wong D. C. J. Rensing K. H. Laflamme-Yonkman J. (2020). Cannabis glandular trichomes alter morphology and metabolite content during flower maturation. *Plant J.* 101 37–56. 10.1111/tpj.14516 31469934

[B52] Magagnini G. Grassi G. Kotiranta S. (2018). The effect of light spectrum on the morphology and cannabinoid content of *Cannabis sativa* L. *Med. Cannabis Cannabinoids* 1 19–27. 10.1159/000489030 34676318 PMC8489345

[B53] Mehmedic Z. Chandra S. Slade D. Denham H. Foster S. Patel A. S. (2010). Potency trends of Δ9-THC and other cannabinoids in confiscated cannabis preparations from 1993 to 2008. *J. Forensic Sci.* 55 1209–1217. 10.1111/j.1556-4029.2010.01441.x 20487147

[B54] Mikuriya T. H. (1969). Marijuana in medicine: past, present and future. *Calif. Med.* 110 34–40. 4883504 PMC1503422

[B55] Nahtigal I. Blake A. Hand A. Florentinus-Mefailoski A. Haleh H. Friedberg J. (2016). The pharmacological properties of cannabis. *J. Pain Manag.* 9 481–491.

[B56] Naim-Feil E. Pembleton L. W. Spooner L. E. Malthouse A. L. Miner A. Quinn M. (2021). The characterization of key physiological traits of medicinal cannabis (*Cannabis sativa* L.) as a tool for precision breeding. *BMC Plant Biol.* 21:294. 10.1186/s12870-021-03079-3072 34174826 PMC8235858

[B57] Nguyen T. T. Slaughter D. C. Max N. Maloof J. N. Sinha N. (2015). Structured light-based 3D reconstruction system for plants. *Sensors* 15 18587–18612. 10.3390/s150818587 26230701 PMC4570338

[B58] Pacifico D. Miselli F. Micheler M. Carboni A. Ranalli P. Mandolino G. (2006). Genetics and marker-assisted selection of the chemotype in *Cannabis sativa* L. *Mol. Breed.* 17 257–268. 10.1007/s11032-005-5681-x

[B59] Pain S. (2015). A potted history. *Nature* 525 S10–S11. 26398731 10.1038/525S10a

[B60] Parker K. A. Di Mattia A. Shaik F. Cerón Ortega J. C. Whittle R. (2019). Risk management within the cannabis industry: building a framework for the cannabis industry. *Financ. Mark. Institutions Instruments* 28 3–55. 10.1111/fmii.12104

[B61] Parker L. A. Mechoulam R. Schlievert C. (2002). Cannabidiol, a non-psychoactive component of cannabis and its synthetic dimethylheptyl homolog suppress nausea in an experimental model with rats. *Neuroreport* 13 567–570. 10.1097/00001756-200204160-200204166 11973447

[B62] Pisanti S. Bifulco M. (2017). Modern history of medical cannabis: from widespread use to prohibitionism and back. *Trends Pharmacol. Sci.* 38 195–198. 10.1016/j.tips.2016.12.002 28095988

[B63] Potter D. J. (2013). A review of the cultivation and processing of cannabis (*Cannabis sativa* L.) for production of prescription medicines in the UK. *Drug Test. Anal.* 6 31–38. 10.1002/dta.1531 24115748

[B64] R Core Team (2020). *R: A Language and Environment for Statistical Computing.* Vienna: R Core Team.

[B65] Radwan M. M. Wanas A. S. Chandra S. ElSohly M. A. (2017). “Natural cannabinoids of cannabis and methods of analysis,” in *Cannabis sativa L. - Botany and Biotechnology*, eds Chandra S. Lata H. ElSohly M. A. (Berlin: Springer International Publishing), 161–182. 10.1007/978-3-319-54564-6_7

[B66] Raman V. Lata H. Chandra S. Khan I. A. ElSohly M. A. (2017). “Morpho-anatomy of marijuana (*Cannabis sativa* L.),” in *Cannabis sativa L. - Botany and Biotechnology*, eds Chandra S. Lata H. ElSohly M. A. (Berlin: Springer International Publishing), 123–136. 10.1007/978-3-319-54564-6_5

[B67] Reichel P. Munz S. Hartung J. Präger A. Kotiranta S. Burgel L. (2021). Impact of three different light spectra on the yield, morphology and growth trajectory of three different *Cannabis sativa* L. strains. *Plants* 10:1866. 10.3390/plants10091866 34579399 PMC8472666

[B68] Rodriguez-Morrison V. Llewellyn D. Zheng Y. (2021). Cannabis yield, potency, and leaf photosynthesis respond differently to increasing light levels in an indoor environment. *Front. Plant Sci.* 12:646020. 10.3389/fpls.2021.646020 34046049 PMC8144505

[B69] Russo E. B. (2014). “The pharmacological history of cannabis,” in *Handbook of Cannabis*, ed. Pertwee R. (Oxford: Oxford University Press), 12–43. 10.1093/acprof

[B70] Saloner A. Bernstein N. (2021). Nitrogen supply affects cannabinoid and terpenoid profile in medical cannabis (*Cannabis sativa* L.). *Ind. Crops Prod.* 167:113516. 10.1016/j.indcrop.2021.113516

[B71] Samal A. Choudhury S. Das Awada T. (2020). “Part 1: basics,” in *Intelligent Image Analysis for Plant Phenotyping*, eds Samal A. Das Choudhury S. (Boca Raton, FL: CRC Press), 4–66.

[B72] Small E. (2015). Evolution and classification of *Cannabis sativa* (Marijuana, Hemp) in relation to human utilization. *Bot. Rev.* 81 189–294. 10.1007/s12229-015-9157-9153

[B73] Small E. (2017a). “Classification of *Cannabis sativa* L.: in relation to agricultural, biotechnological, medical and recreational utilization,” in *Cannabis sativa L. - Botany and Biotechnology*, eds Chandra S. Lata H. ElSohly M. A. (Berlin: Springer International Publishing), 1–62. 10.1007/978-3-319-54564-6_1

[B74] Small E. (2017b). “Medical marijuana: production,” in *Cannabis: a Complete Guide*, (Boca Raton, FL: CRC Press), 351–370.

[B75] Small E. Pocock T. Cavers P. B. (2003). The biology of Canadian weeds. 119. *Cannabis sativa* L. *Can. J. Plant Sci.* 83 217–237. 10.4141/P02-021

[B76] Sobo E. J. (2017). Parent use of cannabis for intractable pediatric epilepsy: everyday empiricism and the boundaries of scientific medicine. *Soc. Sci. Med.* 190 190–198. 10.1016/j.socscimed.2017.08.003 28865255

[B77] Summers D. J. (2018). “Supply and demand,” in *The Business of Cannabis: New Policies for the New Marijuana Industry*, (California: Praeger), 1–16.

[B78] Tanabata T. Shibaya T. Hori K. Ebana K. Yano M. (2012). SmartGrain: high-throughput phenotyping software for measuring seed shape through image analysis. *Plant Physiol.* 160 1871–1880. 10.1104/pp.112.205120 23054566 PMC3510117

[B79] Tanaka H. Shoyama Y. (1999). Monoclonal antibody against tetrahydrocannabinolic acid distinguishes *Cannabis sativa* samples from different plant species. *Forensic Sci. Int.* 106 135–146. 10.1016/S0379-0738(99)00193-190 10680062

[B80] Tanksley S. D. (2004). The genetic, developmental, and molecular bases of fruit size and shape variation in tomato. *Plant Cell* 16 181–189. 10.1105/tpc.018119 15131251 PMC2643388

[B81] Thomas B. F. ElSohly M. A. (2015). “The botany of *Cannabis sativa* L,” in *The Analytical Chemistry of Cannabis: Quality Assessment, Assurance, and Regulation of Medicinal Marijuana and Cannabinoid Preparations*, eds Thomas B. F. ElSohly M. A. (Amsterdam: Elsevier).

[B82] Yoshioka M. Takenaka S. Nitta M. Li J. Mizuno N. Nasuda S. (2019). Genetic dissection of grain morphology in hexaploid wheat by analysis of the NBRP-Wheat core collection. *Genes Genet. Syst.* 94 35–49. 10.1266/ggs.18-00045 30626760

[B83] Zuardi A. W. (2006). History of cannabis as a medicine: a review. *Rev. Bras. Psiquiatr.* 28 153–157. 10.1590/S1516-44462006000200015 16810401

