# Supplementary material for: Empirical Evaluation of Inflorescences’ Morphological Attributes for Yield Optimization of Medicinal Cannabis Cultivars
Source: Front Plant Sci. 2022 Apr 19;13:858519. doi: 10.3389/fpls.2022.858519 (PMC9063709; doi:10.3389/fpls.2022.858519)
Supplement: Supplementary file 1 [file Image_1.pdf]

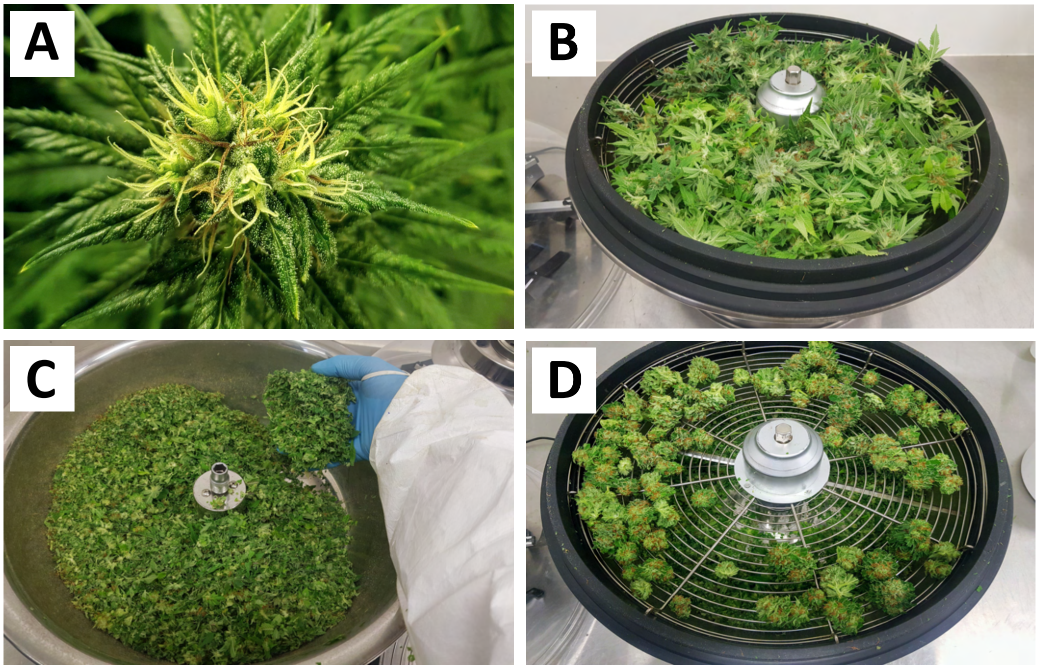

Figure S1: Plant processing and inflorescence preparation for image analysis evaluation. A) Inflorescence with stigmas transitioning to brown shaded colour, B) Raw inflorescence material over the leaf trimmer, C) Trimmed by-product , D) Processed inflorescence material.
