# Supplementary material for: Empirical Evaluation of Inflorescences’ Morphological Attributes for Yield Optimization of Medicinal Cannabis Cultivars
Source: Front Plant Sci. 2022 Apr 19;13:858519. doi: 10.3389/fpls.2022.858519 (PMC9063709; doi:10.3389/fpls.2022.858519)
Supplement: Supplementary file 2 [file Image_2.pdf]

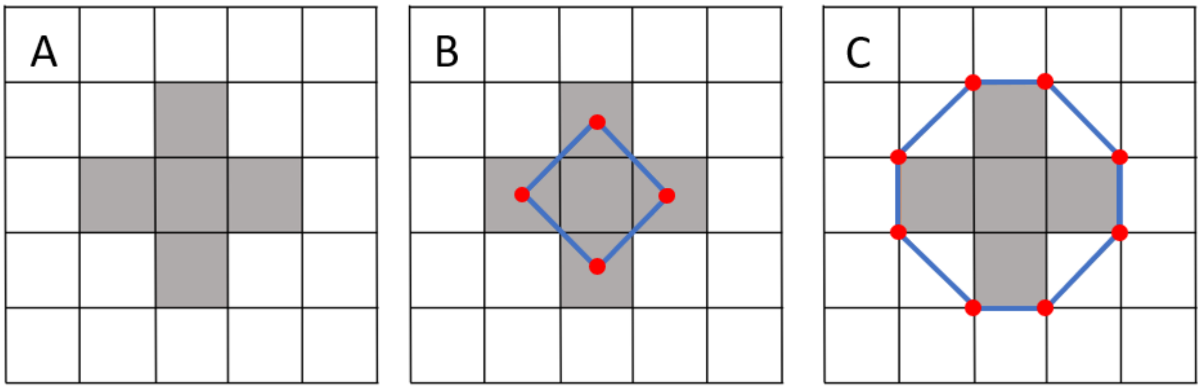

Figure S2: Correcting pixel location for geometric area determination. A) A cross object consisting of 5 pixels. B) The outer 4 pixels that form the convex hull (CH) point set are highlighted with central red dots and the associated polygon is shown in blue. Note, however, if the area of this polygon is calculated, using the raw pixel positions as highlighted, an incorrect value of 2 will be obtained. C) Expanding the initial CH points to the appropriate pixel corner locations a 2nd hull set is created, as highlighted by red points, and when the area of the associated polygon, shown in blue, the correct hull area of 7 will be obtained.
