# Supplementary material for: Empirical Evaluation of Inflorescences’ Morphological Attributes for Yield Optimization of Medicinal Cannabis Cultivars
Source: Front Plant Sci. 2022 Apr 19;13:858519. doi: 10.3389/fpls.2022.858519 (PMC9063709; doi:10.3389/fpls.2022.858519)
Supplement: Supplementary file 3 [file Image_3.pdf]

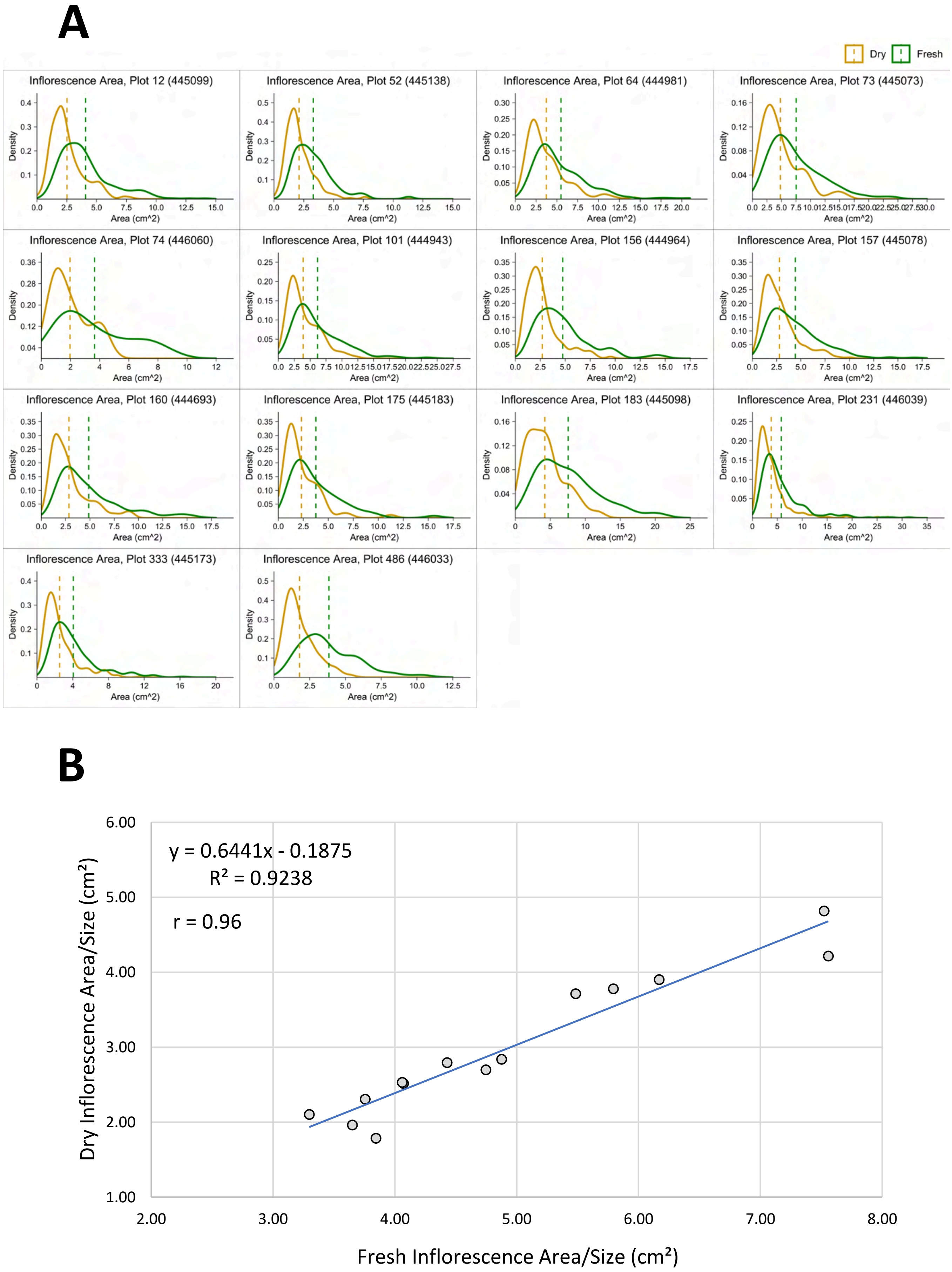

<u>Figure S3-1</u>: Variation in Inflorescence Size (IS) under dry and fresh states. A) Frequency distributions of IS across 14 selected plants. Yellow and green density lines indicate the IS distribution under dry and fresh states, respectively. The average IS is indicated by dashed lines. B) A linear regression between the fresh and dry IS mean across 14 plants. The regression trendline equation and its R^2 value are located in the top left corner, r indicates the correlation coefficient value calculated between the examined parameters.

A

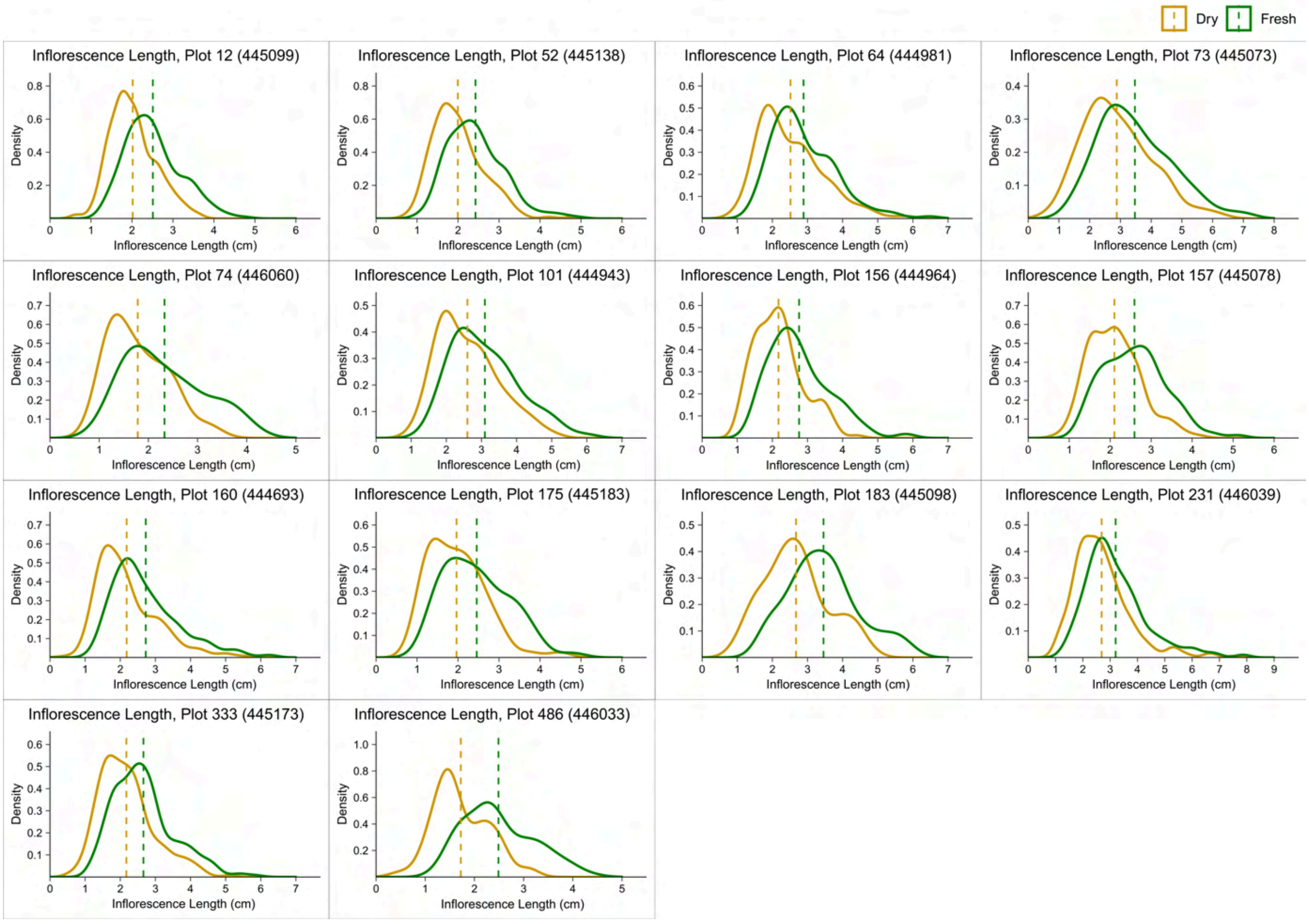

B

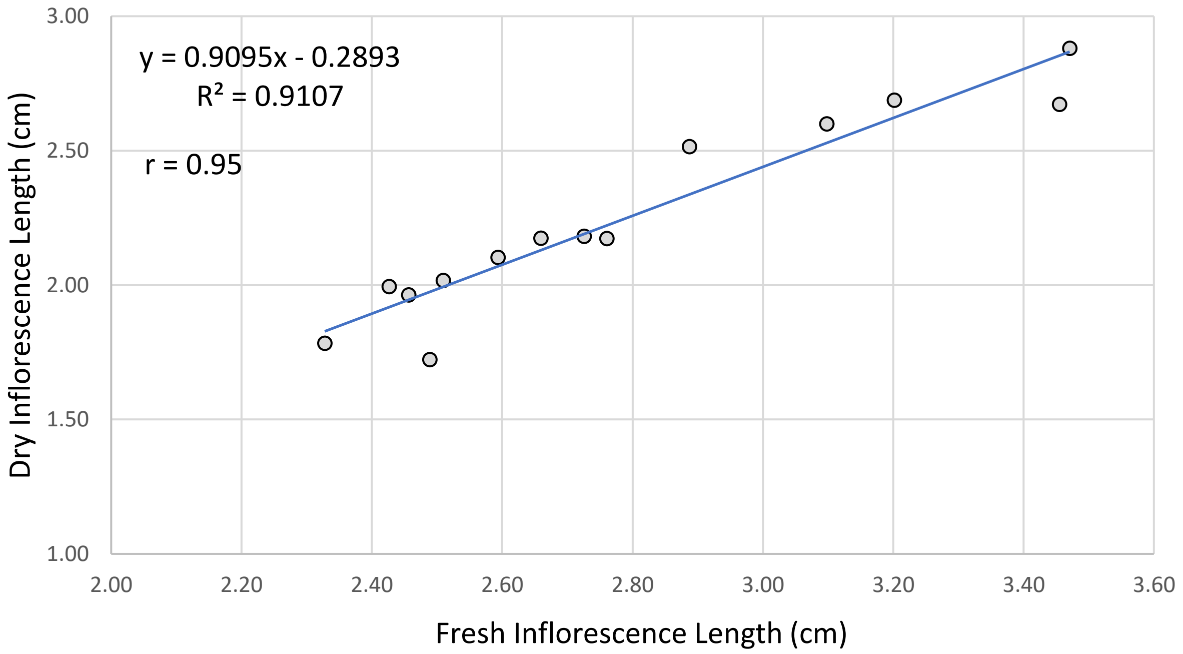

Figure S3-2: Variation in Inflorescence Length (IL) under dry and fresh states. A) Frequency distributions of IL across 14 selected plants. Yellow and green density lines indicate the IL distribution under dry and fresh states, respectively. The average IL is indicated by dashed lines. B) A linear regression between the fresh and dry IL mean across 14 plants. The regression trendline equation and its R^2 value are located in the top left corner, r indicates the correlation coefficient value calculated between the examined parameters.

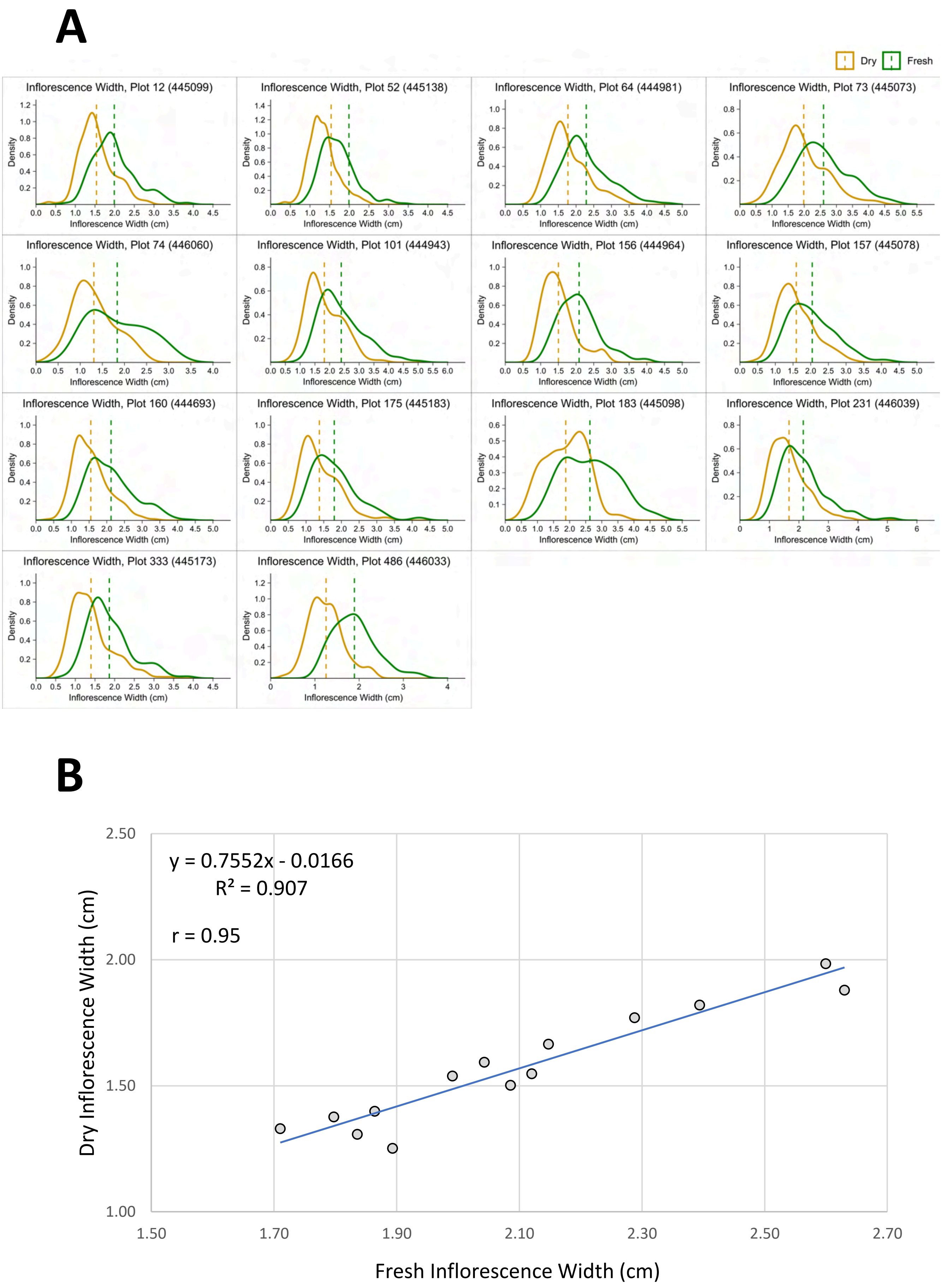

Figure S3-3: Variation in Inflorescence Width (IW) under dry and fresh states. A) Frequency distributions of IW across 14 selected plants. Yellow and green density lines indicate the IW distribution under dry and fresh states, respectively. The average IW is indicated by dashed lines. B) A linear regression between the fresh and dry IW mean across 14 plants. The regression trendline equation and its R^2 value are located in the top left corner, r indicates the correlation coefficient value calculated between the examined parameters.

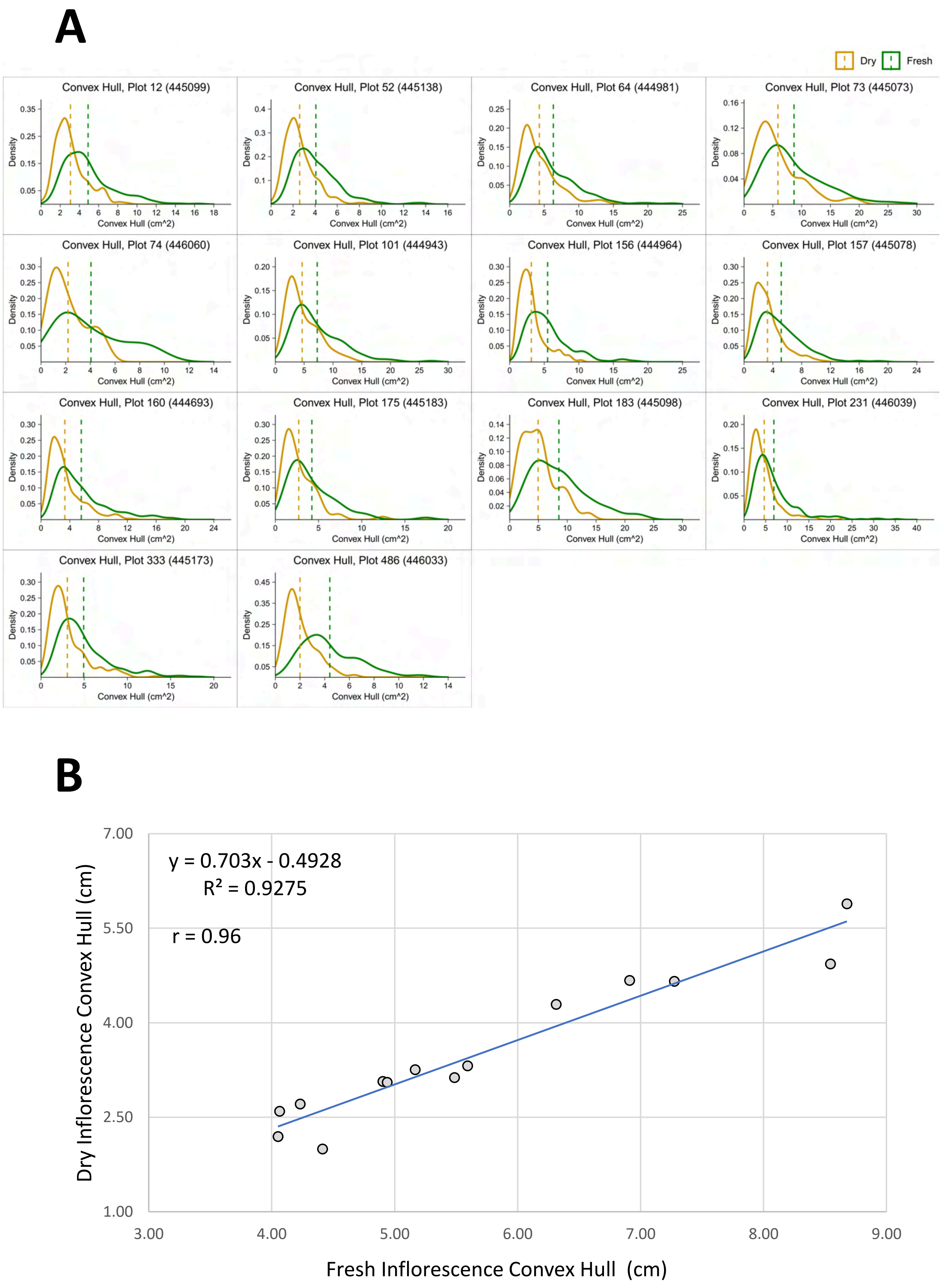

Figure S3-4: Variation in inflorescence Convex Hull (CH) under dry and fresh states. A) Frequency distributions of CH across 14 selected plants. Yellow and green density lines indicate the CH distribution under dry and fresh states, respectively. The average CH is indicated by dashed lines. B) A linear regression between the fresh and dry CH mean across 14 plants. The regression trendline equation and its R^2 value are located in the top left corner, r indicates the correlation coefficient value calculated between the examined parameters.

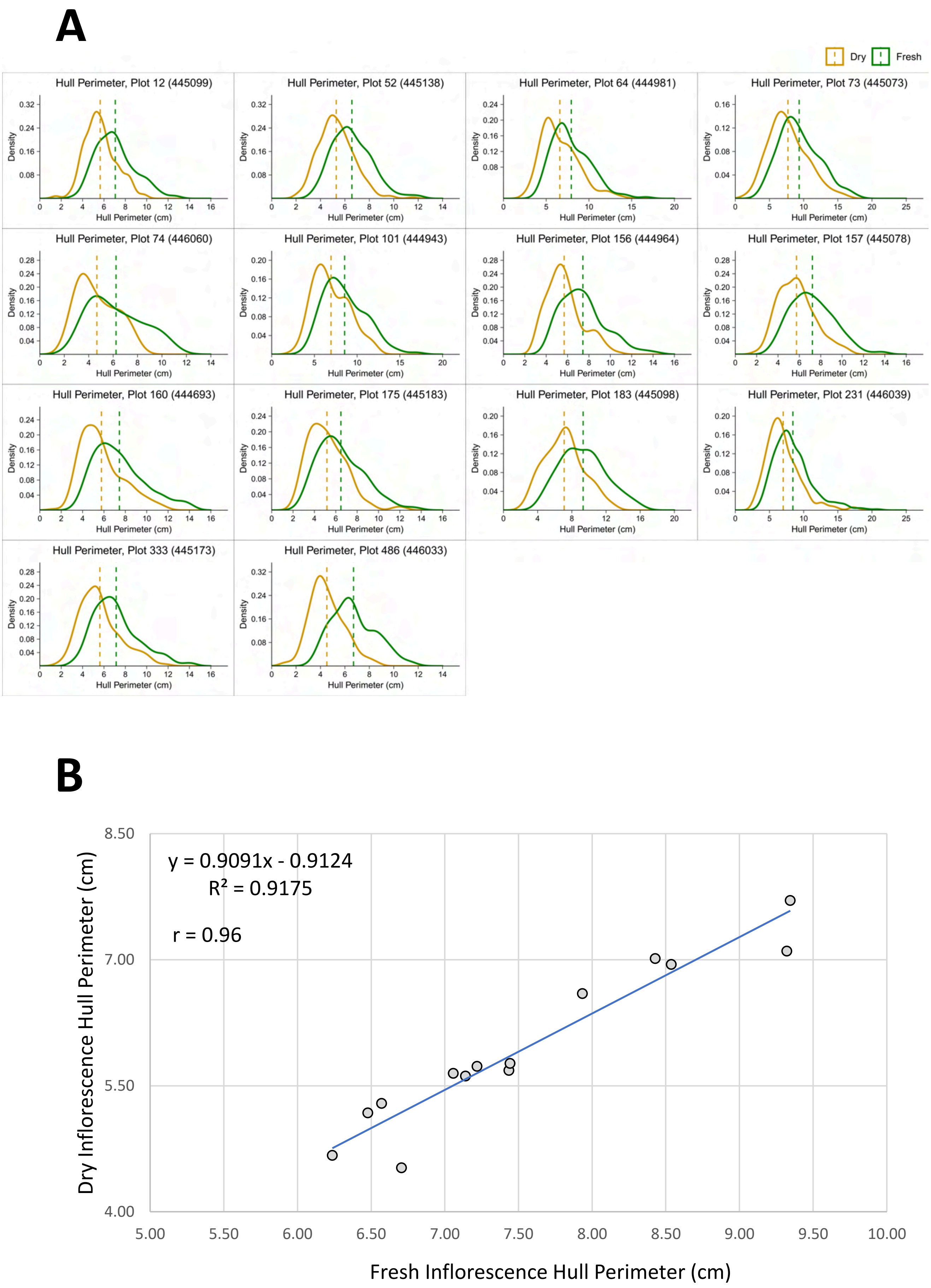

Figure S3-5: Variation in inflorescence Hull Perimeter (HP) under dry and fresh states. A) Frequency distributions of HP across 14 selected plants. Yellow and green density lines indicate the HP distribution under dry and fresh states, respectively. The average HP is indicated by dashed lines. B) A linear regression between the fresh and dry HP mean across 14 plants. The regression trendline equation and its R^2 value are located in the top left corner, r indicates the correlation coefficient value calculated between the examined parameters.
